# Arylgold nanoclusters: Phenyl-stabilized Au_44_ with thermal-controlled NIR single/dual-channel phosphorescence

**DOI:** 10.1126/sciadv.adm6928

**Published:** 2024-02-14

**Authors:** Wei-Dan Si, Chengkai Zhang, Meng Zhou, Zhi Wang, Lei Feng, Chen-Ho Tung, Di Sun

**Affiliations:** ^1^ School of Chemistry and Chemical Engineering, State Key Laboratory of Crystal Materials, Shandong University, Jinan 250100, People’s Republic of China.; ^2^ Hefei National Research Center for Physical Sciences at the Microscale, Department of Chemical Physics, University of Science and Technology of China, Hefei 230026, Anhui, People’s Republic of China.

## Abstract

Arylation of gold holds paramount importance in the domain of organometallic chemistry; however, the exploration of arylgold nanoclusters remains in its infancy primarily due to the synthetic challenge. Here, we present a facile and effective arylation strategy to directly synthesize two arylgold nanoclusters (**Au44a** and **Au44b**), by using tetraarylborates, capable of transferring aryl fragments to metal centers. X-ray crystallography reveals that both Au_44_ nanoclusters contain an Au_44_ kernel co-protected by six aryl groups, two tetrahydrothiophene, and 16 alkynyl-ether ligands, the latter is generated in situ through Williamson ether reaction during the assembly processes. Notably, Au_44_ nanoclusters exhibit near-infrared (NIR) phosphorescence (λ_max_ = 958 nm) and microsecond radiative relaxation at ambient condition, which is a thermal-controlled single/dual-channel phosphorescent emission revealed by temperature-dependent NIR, time-resolved emission, and femtosecond/nanosecond transition absorption spectra. This work represents a breakthrough in using aryl as protective ligands for the construction of gold nanoclusters, which is poised to have a transformative impact on organometallic nanoclusters.

## Teaser

Arylated gold nanoclusters exhibit impressive thermal-controlled single/dual-channel NIR phosphorescent emission.

## INTRODUCTION

Arylgold complexes play a vital role in the field of organometallic chemistry, with ubiquitous applications as synthetic intermediates, emissive materials, and catalysts (*1*–*4*). Consequently, numerous research teams worldwide have dedicated substantial efforts to the development of efficient methods for incorporating aryl groups into gold complexes (*2*, *5*–*8*). Aryllithium, Grignard reagents, organomercury, and organothallium compounds are frequently used as common aryl transfer agents for the preparation of arylgold complexes (*5*, *6*). However, because of the flammable or toxic nature of these arylating agents, the reaction process often requires relatively harsh conditions. Subsequently, tetraarylborates and arylboronic acids have emerged as highly favored options for transferring aryl fragments to gold atoms due to their easy availability and cleavable C─B bonds, even under a mild condition (*8*–*11*). With the advent of high-throughput synthetic methods, the quantity and diversity of arygold complexes have expanded substantially, enabling comprehensive investigations of their properties. However, the progress in the explorations of arylgold nanoclusters has been sluggish and even stagnant.

**Fig. 1.**
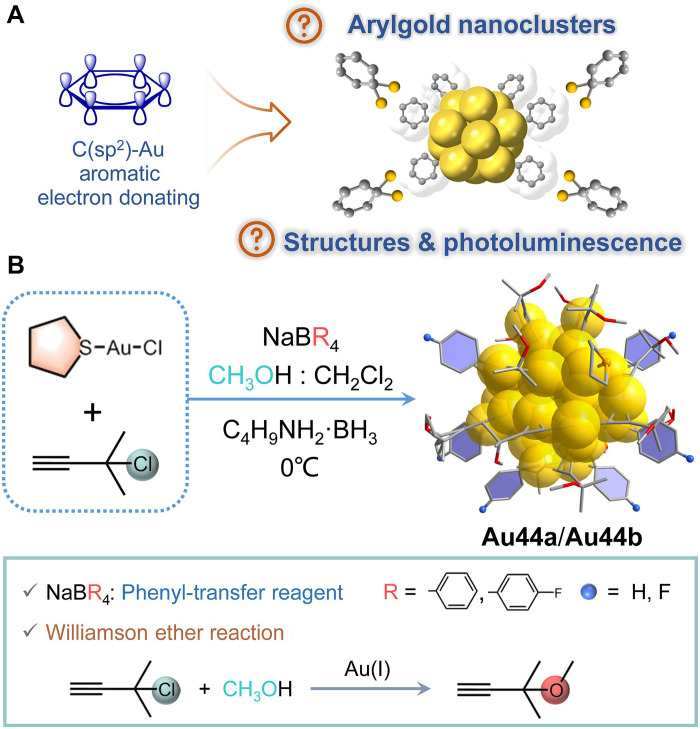
Significance and strategy for accessing arylgold nanoclusters. (**A**) The guiding ideology of this work. (**B**) Schematic representation of the synthesis of **Au44a** and **Au44b**.

Ligand-protected gold nanoclusters with atomically precise compositions have attracted increasing attention, owing to their diverse structures, unique physical and chemical properties and widespread applications in various fields (*12*–*14*). Surface ligands have been well documented to play a crucial role in determining the size, shape, and properties of gold nanoclusters (*15*–*18*). Thus, since the advent of the first case of phosphine-stabilized gold nanoclusters in 1969 (*19*), researchers have explored a succession of organic ligands, including phosphines, thiols, alkynyls, and nitrogen donors to protect gold nanoclusters (*12*, *20*–*25*). More recently, N-heterocyclic carbenes (NHCs), known for their strong electron-donating capabilities, have been developed for the stabilization and functionalization of gold nanoclusters (*26*–*30*). Despite these advancements, phenyl group as one of the most basic aromatic hydrocarbons has been known to have the potential for constructing gold complexes with diverse properties, which not only can directly interact with gold atoms by π-system but also can coordinate with gold atoms by μ- and μ_2_-bridging Au─C bonds after deprotonation (*9*–*11*). Thus, aryl ligands should also be excellent candidates for stabilizing gold nanoclusters and achieving potential photoluminescence (PL) properties. Regrettably, there is now limited knowledge about arylgold nanoclusters, and, to our knowledge, only one small cluster of μ-aryl Au_10_ was found so far (*10*). This scarcity of examples severely hampers the development of arylgold nanoclusters. Although arylation on gold nanocluster is fraught with difficulties and obstacles, it is highly desirable due to its infinite potential for regulating the structures and properties of gold nanoclusters (Fig. 1A).

Here, we targeted to develop a facile and effective synthetic strategy to construct arylgold nanoclusters using tetraarylborates as aryl transfer agents. Two aryl-protected Au_44_ nanoclusters, Au_44_[CH_3_O(CH_3_)_2_C≡C]_16_(C_6_H_5_)_6_(tht)_2_·CH_2_Cl_2_·H_2_O (**Au44a**) and Au_44_[CH_3_O(CH_3_)_2_C≡C]_16_(*p*-C_6_H_4_F)_6_(tht)_2_·3CH_2_Cl_2_·H_2_O (**Au44b**) (tht = tetrahydrothiophene), have been successfully obtained via a one-pot reaction. **Au44a** in 2-methyltetrahydrofuran (2-Me-THF) exhibits phosphorescence (PH) emission in the near-infrared (NIR) region (λ_em_ = 958 nm) with a peculiar temperature-dependent emission behavior. Steady- and transient-state studies on **Au44a** showed that the PH of **Au44a** contains two triplet excited states (T_1_ and T_1_′) with a small activation barrier that can be overcome at temperature exceeding 100 K, and, furthermore, thermal-controlled switching between single and dual channels PH emission is realized. This study not only represents the first direct synthesis of aryl-protected gold nanoclusters but also offers crucial insights into the intriguing NIR PL mechanism of gold nanoclusters.

## RESULTS

### Synthesis of Au44a and Au44b

Both **Au44a** and **Au44b** were synthesized by a direct reduction method (Fig. 1B). Briefly, [Au(tht)Cl], 3-chloro-3-methyl-1-butyne [Cl(CH_3_)_2_CC≡CH], NaBPh_4_, and Et_3_N were stirred in a binary solvent of CH_3_OH and CH_2_Cl_2_ at 0°C for 10 min, to which a freshly prepared solution of tert-butylaminebrane (C_4_H_9_NH_2_·BH_3_) was added. This reaction continued at 0°C for 16 hours, during which time the color of solution changed from pale yellow to dark brown. After 2 days, black crystals of **Au44a** were crystallized from the reaction mother liquor in the dark at 4°C. The phase purity of **Au44a** was confirmed by the powder x-ray diffraction (PXRD) (fig. S1). Notably, the success in the synthesis of **Au44a** is highly dependent on two critical factors: (i) the release of phenyl from NaBPh_4_ and (ii) the in situ generation of 3-methoxy-3-methyl-1-butyne [CH_3_O(CH_3_)_2_C≡CH] ligands via the Williamson ether reaction. First, because of the C─B bond cleavage, NaBPh_4_ serves as a special phenyl-transfer reagent to provide unique Ph^−^ ligands to stabilize gold nanoclusters under a mild condition (*31*, *32*). To the best of our knowledge, this is a very rare example of phenyl transfer to gold nanoclusters. We also tried several derivatives of NaBPh_4_, including NaB(*p*-Ph-F)_4_ [sodium tetrakis(4-fluorophenyl)borate], NaB(C_6_F_5_)_4_ [sodium tetrakis(perfluorophenyl)borate], and NaBArF {sodium tetrakis[3,5-bis(trifluoromethyl)phenyl]borate}. Only upon replacing NaBPh_4_ with NaB(*p*-Ph-F)_4_, an analog **Au44b** can be isolated. In addition, we performed control experiments without adding NaBPh_4_ or by replacing NaBPh_4_ with benzene and isolated a distinct nanocluster, Au_22_[CH_3_O(CH_3_)_2_C≡C]_18_ (**Au22**) (fig. S2), with a similar kernel to that of Au_22_(*^t^*BuC≡C)_18_ (*33*). From these results, it is clear that the steric hindrance of aryl dictates its coordination on the surface of gold nanoclusters (*34*). Second, the introduced Cl(CH_3_)_2_CC≡CH ligands undergo an in situ Williamson ether reaction together with CH_3_OH, leading to the formation of CH_3_O(CH_3_)_2_C≡CH (*35*). Furthermore, we also performed gas chromatography–mass spectrometry (MS) analysis to confirm their originations. As shown in fig. S3, after reaction carrying 16 hours, CH_3_O(CH_3_)_2_C≡CH [mass/charge ratio (*m/z*) = 83] and benzene (*m/z* = 78) can be easily identified, accompanied by superfluous Cl(CH_3_)_2_CC≡CH and by-product biphenyl. In contrast, the CH_3_OH solution of pure Cl(CH_3_)_2_CC≡CH or NaBPh_4_ showed only single signal (fig. S4), which are ascribed to Cl(CH_3_)_2_CC≡CH itself and benzene, respectively, indicating the in situ generation of CH_3_O(CH_3_)_2_C≡CH and the ability of NaBPh_4_ to release phenyl. More characterization (figs. S5 and S6) can be found in the Supplementary Materials.

### Crystal structures of Au44a and Au44b

**Fig. 2.**
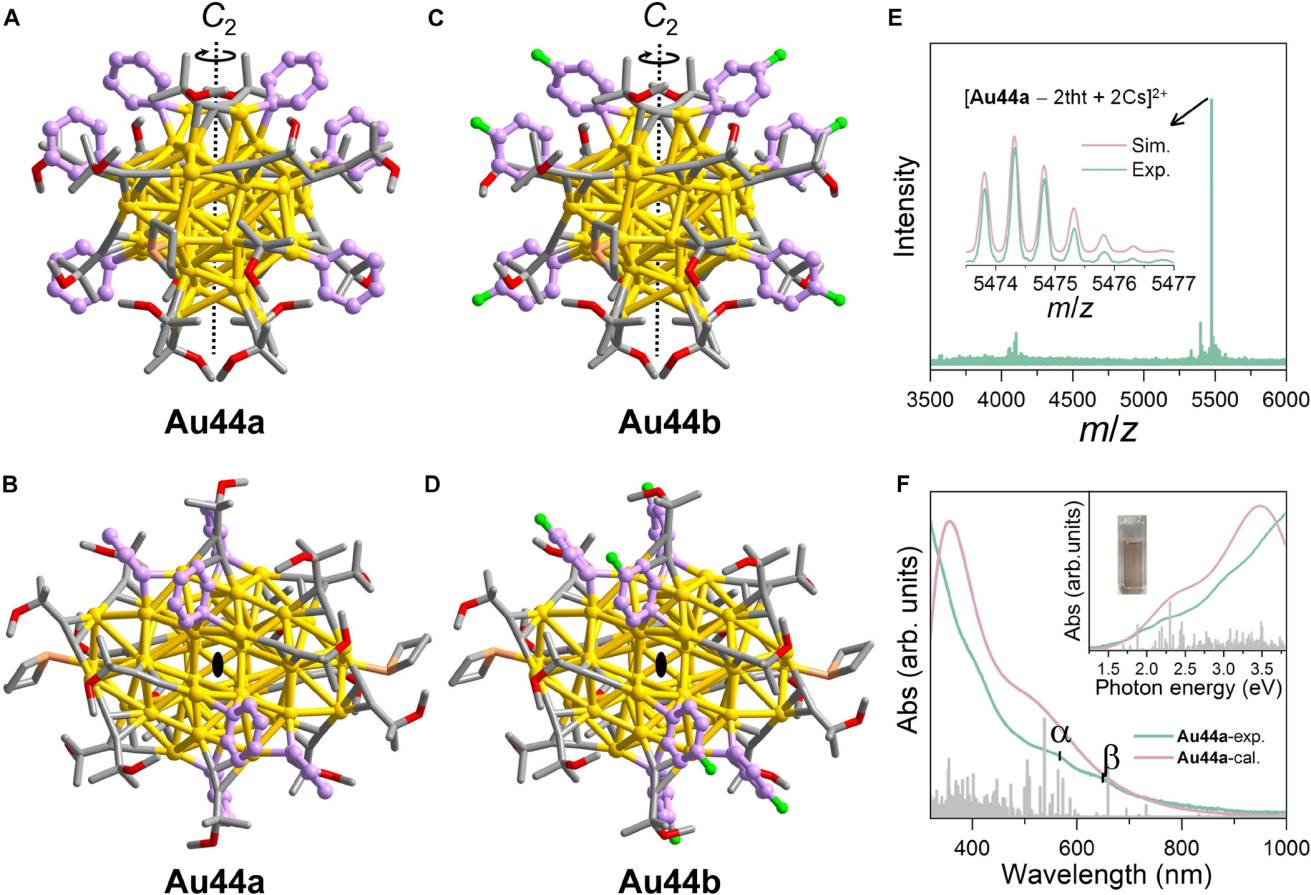
Characterization of the Au_44_ nanoclusters. (**A** and **B**) Front and top views of total molecular structure of **Au44a**. (**C** and **D**) Front and top views of total molecular structure of **Au44b**. (**E**) ESI mass spectrum of **Au44a** dissolved in a mixed solution of CH_2_Cl_2_ and CH_3_OH in positive-ion mode. Inset: The experimental (green trace) and simulated (pink trace) isotopic patterns. (**F**) Experimental (green trace) and calculated (pink trace) absorption spectra of **Au44a**. All hydrogen atoms are omitted for clarity. Color codes: golden, Au; orange, S; red, O; green, F; and purple, gray, C.

The structures of **Au44a** and **Au44b** were determined by single-crystal x-ray diffraction at 100 K, which revealed that both **Au44a** and **Au44b** crystallized in the monoclinic space group *C*2/*c* (table S1). Because **Au44a** and **Au44b** are isostructural and differ only in the protective ligands (Ph^−^ versus *p*-F-Ph^−^) (Fig. 2, A to D), we take **Au44a** as an example for structural analysis in detail below. **Au44a** contains 44 gold atoms and 16 CH_3_O(CH_3_)_2_C≡C^−^, six Ph^−^, and two tht ligands and has a *C*_2_ symmetry. No counterions were observed in their crystal lattices, indicating the charge neutrality of nanoclusters. Electrospray ionization (ESI)–MS was also used to confirm the electroneutral nature of **Au44a**. Upon addition of cesium acetate (CsOAc) to form positively charged adducts, a dominant peak centered at *m/z* = 5474.3183 was observed, corresponding to [**Au44a** − 2tht + 2Cs]^2+^ (calculated *m/z* = 5474.3215) (Fig. 2E). The total number of valence electrons can be determined to be 22 (22 = 44 −16 − 6) (*36*), which corresponds to the superatomic electron configuration of 1S^2^1P^6^1D^10^2S^2^2P^2^ but is not in the magic number series of the spherical jellium model (*37*). The structural and electronic properties of **Au44a** and **Au44b** were further examined by density functional theory (DFT) (Fig. 2F and figs. S7 to S9), based on their crystal structures.

**Fig. 3.**
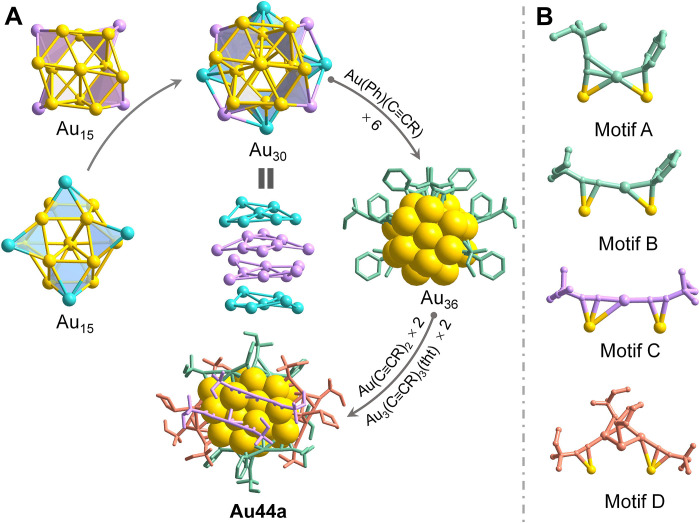
Structural anatomy of Au44a. (**A**) The structure of **Au44a** composed of an Au_30_ kernel that can be regarded as the fusion of two Au_15_ unit, or a layer-by-layer (Au_7_-Au_8_-Au_8_-Au_7_) self-assembly pattern, six Ph─Au─C≡C(CH_3_)_2_OCH_3_ (cyan), 2CH_3_O(CH_3_)_2_C≡C─Au─C≡C(CH_3_)_2_OCH_3_ (purple), and two Au_3_(tht)[CH_3_O(CH_3_)_2_C≡C]_3_ (orange). (**B**) Four different binding motifs: Motif A, Motif B, Motif C, and Motif D.

Figure 3 shows the molecular structure of **Au44a**. It has a unique Au_30_ kernel, which is composed of two Au_15_ units joined together (fig. S10A). Each Au_15_ kernel comprises an Au_10_ kernel surrounded by four peripheral gold atoms, two of which cap on two triangular faces, while the other two cap on two quadrangular faces of Au_10_ kernel (fig. S10B). The Au─Au bond lengths in Au_15_ kernel range from 2.678 to 3.211 Å, giving an average value of 2.837 Å (shorter than the distance of 2.88 Å in bulk gold), indicating the strong interaction between Au atoms in Au_15_ kernel (*38*). The distorted quadrangular-star Au_8_ faces of two Au_15_ units are positioned face to face (Fig. 3A and fig. S10A) and connected by multiple Au─Au bonds between 2.782 and 3.254 Å, resulting in a 1.82% extension of the average kernel distance. Alternatively, the Au_30_ kernel can be regarded to have a layer-by-layer (Au_7_-Au_8_-Au_8_-Au_7_) self-assembly pattern. Such a kernel is completely different from alkynyl- and NHC-protected 44-gold nanoclusters in previous reports (*28*, *39*), which have face-centered cubic–type Au_36_ and decapped vertex-sharing biicosahedral Au_23_ kernel, respectively, implying that ligands play a crucial role in dictating the nanocluster structures.

Notably, the most intriguing structural feature in **Au44a** is the interfacial bonding staples involving Ph^−^ ligands on the periphery of Au_30_ kernel. As portrayed in Figs. 3 and 6, Ph^−^ ligands act as a kind of competent passivating agent, each adopting a μ_2_-bridging mode to coordinate with two gold atoms, which is the similar to the μ_2_ alkynyl or thiolate ligands in gold nanoclusters. Each Ph^−^ ligand combines with one CH_3_O(CH_3_)_2_C≡C^−^ to further form unprecedented Ph─Au─C≡C (CH_3_)_2_OCH_3_ staple motifs (two motif A and four motif B) located on the Au_30_ kernel (Fig. 3B). The alkynyl ligands in motif A and motif B both adopt μ_2_-η_σ_^1^,η_π_^2^ coordination mode, but they ligate gold atom from the Au_30_ kernel through different binding modes, σ and π bonds, respectively (Fig. 3B). In addition, there are still two linear CH_3_O(CH_3_)_2_C≡C─Au─C≡C(CH_3_)_2_OCH_3_ dimeric staple motifs (motif C) at the bottom of Au_30_ kernel (Fig. 3B). Two special staple motifs, Au_3_(tht)[CH_3_O(CH_3_)_2_C≡C]_3_ (motif D), are symmetrically distributed around the Au_30_ kernel. They can alternatively be seen as two V-shaped CH_3_O(CH_3_)_2_C≡C─Au─C≡C[(CH_3_)_2_OCH_3_]─Au─C≡C(CH_3_)_2_OCH_3_ trimeric staple motifs (motif E) (fig. S11) as well as two tht─Au─ sealing the left and right ends of nanocluster. The Au─C(sp) bonds between CH_3_O(CH_3_)_2_C≡C^−^ ligands and gold atoms give an average value of 2.17 Å with σ and π bonds lying in the range of 1.87 to 2.00 Å and 2.02 to 2.58 Å, respectively. In contrast, the average Au─C(sp^2^) distance between Ph^−^ and gold atoms in **Au44a** is 2.14 Å (range. 2.12 to 2.15 Å) (table S2), which is obviously longer than those of NHC-stabilized gold nanoclusters (*26*–*30*) but comparable to those of alkynyl-protected gold nanoclusters (*40*, *41*), indicating relatively strong bonding interactions. The average Au─C(sp^2^)─Au angles range from 78.3° to 79.0°, much smaller than the Au─C(sp)─Au angles in the range of 88.3° to 111.2°.

Contributed by the unique metal-organic interface and surface ligand arrangement, substantial intra/intercluster interactions exist in Au_44_ nanoclusters. Each phenyl rings on the surface of **Au44a** can interact with adjacent phenyl rings or methyl group of alkynyl ligands to form strong C_(Ph)_─H···π_(Ph)_ (2.66 Å) or C_(Me)_─H···π_(Ph)_ (3.06 to 3.39 Å) intracluster interactions (fig. S12). In the crystal lattice of **Au44a**, along the *c* axis (fig. S13), adjacent layers exhibit abundant C_(Me)_─H···π_(C≡C)_, C_(Me)_─H···O, and H···H interactions, and, in each layer, C_(Me)_─H···π_(Ph)_ and weak H···H interactions are also found between neighboring Au_44_ nanoclusters (fig. S14). In contrast, although **Au44b** also adopts similar packing pattern and interactions to **Au44a**, the presence of fluorine-rich *p*-F-Ph^−^ ligands brings two unique intercluster interactions that are not observed in **Au44a**: (i) C─H···F hydrogen bonds between *p*-F-Ph^−^ and tht or methoxyl group of alkynyl ligands (*d*_H···F_, 2.66 or 2.54 Å) and (ii) C─F···Au (*d*_F···Au_, 2.95 Å) metal-involving halogen bond (XB) interactions based on International Union of Pure and Applied Chemistry (IUPAC) criteria for XB (figs. S15 and 16) (*42*). Notably, such a short F···Au contact is very rare in the self-assembly of gold nanoclusters.

### Nature of the NIR PH for Au_44_

**Fig. 4.**
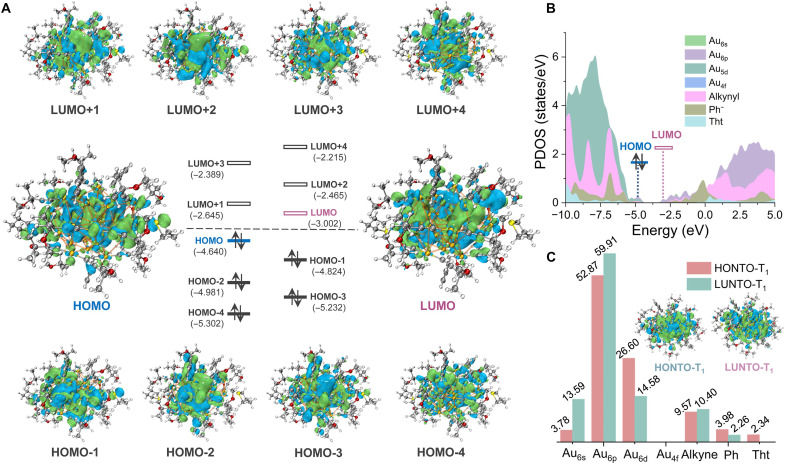
Calculated electronic structure of Au44a. (**A**) Isosurface plots (isodensity value = 0.02 arbitrary units) of molecular orbitals of **Au44a** and their corresponding energy levels. (**B**) Projected electronic density of states (PDOS) of **Au44a**. (**C**) The contribution (%) of gold atoms and ligands to highest occupied and lowest occupied natural transition orbitals of **Au44a**. HOMO, highest occupied molecular orbital; LUMO, lowest unoccupied molecular orbital.

The photophysical properties of Au_44_ nanoclusters were studied in solution prepared by picking up single crystals and dissolving them in 2-Me-THF to ensure the purity. Because **Au44a** and **Au44b** show a similar steady-state absorption and PL spectra (fig. S17), **Au44a** was selected as a representative for further analysis. The slight bathochromic shift of characteristic peaks in the absorption and PL spectra of **Au44b** is attributed to electron-withdrawing inductive effect of F atoms present in the aryl ligands (*43*). The optical absorption spectrum of **Au44a** is shown in Fig. 2F, with two major bands, α and β, centered at 554 nm (2.24 eV) and 634 nm (1.96 eV), respectively, along with a very broad low energy absorption between λ_abs_ = 700 and 1000 nm. According to our time-dependent (TD)-DFT calculations (Fig. 4), the calculated HOMO-LUMO gap was determined to be 1.638 eV, which closely matches the experimental value of 1.578 eV. The band α mainly arises from excitation to L_(ph+alkynyl+Tht)_MCT states with MM_(Au:d-sp)_CT admixtures, while the low energy absorption band β is attributed to the transitions of dominant MM_(Au:d-sp)_CT character with a small proportion of L_(ph+alkynyl+Tht)_MCT states.

**Fig. 5.**
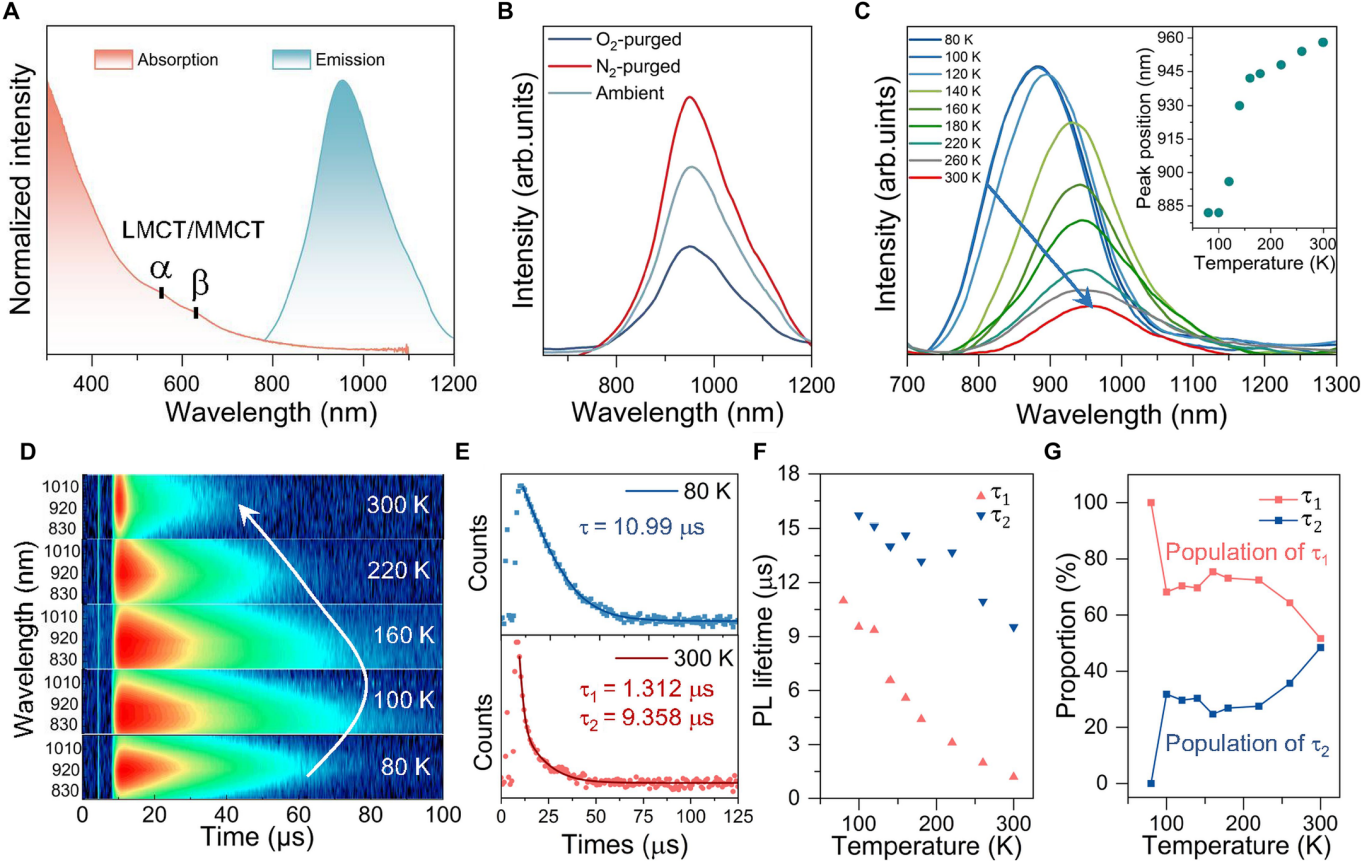
NIR PH property of Au44a. (**A**) Steady-state absorption and NIR emission spectra of **Au44a** in 2-Me-THF. (**B**) NIR emission spectra of **Au44a** under O_2_-purged, N_2_-purged, and ambient condition; λ_ex_ = 365 nm. (**C**) Temperature-dependent NIR emission spectra of **Au44a** in 2-Me-THF. (**D**) Time-resolved emission spectra of **Au44a** at different temperatures. (**E**) Decay curves of **Au44a** at 80/300 K. (**F** and **G**) The contributions of τ_1_ and τ_2_ as a function of temperature.

The PL emission of **Au44a** in 2-Me-THF is broad with a large full width at half maximum (FWHM) of ~180 nm and a maximum at λ_em_ = 958 nm (Fig. 5A), apparently from the coupling of MM_(Au:d-sp)_CT and LM_(Au)_CT states. Notably, **Au44a** exhibits a bi-exponential PL decay, consisting of two microsecond-magnitude components with nearly identical fractional populations (τ_1_, 1.20 μs, *f* = 51.62%; τ_2_, 9.53 μs, *f* = 48.38%), suggesting that **Au44a** acts as a triplet photosensitizer with two radiative processes (*41*, *44*). For verification of the triplet character, the NIR PL spectra of **Au44a** in N_2_-purged and O_2_-saturated 2-Me-THF were measured, as displayed in Fig. 5B. A conspicuous quenching of PL was observed for **Au44a** under pure O_2_ condition, while an enhanced PL signal was detected under N_2_ condition. Meanwhile, the N_2_-purged and O_2_-saturated solution of **Au44a**, respectively, gave longer and shorter average lifetimes of 8.97 and 8.16 μs (Table 1 and fig. S18). The absorption spectra of **Au44a** before and after purging with O_2_ exhibit a consistency (fig. S19), suggesting that **Au44a** did not undergo oxidation when exposed to the oxygen environment. In addition, a rapid decomposition of 1,3-diphenylisobenzofuran was observed in the presence of **Au44a**, as monitored by ultraviolet-visible (UV-vis) absorption spectra at ~411 nm in air (fig. S20), suggesting that **Au44a** is a promising photosensitizer for singlet oxygen (^1^O_2_) generation. These features are essential for evaluating the ^1^O_2_ generation contributed by **Au44a** under Xe irradiation, further validating its PH nature. The Φ_Ph_ value of this sample was determined to be 0.8% using the relative method. Excitation- and polarity-dependent PL measurements were carried out to investigate the nature of ground and excited states of NIR PH in **Au44a**. In terms of the ground state, the PL excitation (PLE) spectrum of **Au44a** at 958-nm emission wavelength exhibits a similar spectral profile to its absorption spectrum (fig. S21), and no emission wavelength dependence was observed for the two-dimensional PL/PLE map (fig. S22) excited between 300 and 600 nm, suggesting that the NIR PH of **Au44a** origins from one emitter (*45*). For the excited state, even dissolved in three other solvents (CH_2_Cl_2_, CHCl_3_, and toluene) with different polarity, the profiles of the absorption, emission spectra, and excited decay times of **Au44a** are almost same (figs. S23 and 24). These results reflect that the excited states of **Au44a** are less affected by exterior solvent polarity and imply that the emissive states of **Au44a** primarily originate from the cluster center rather than the surface ligand layer that are susceptible to external environmental factors. This deduction was further confirmed by TD-DFT simulation (Fig. 4C). For the natural transition orbital analysis of S_0_-T_1_ excitations, the situations in **Au44a** show similar transition compositions to the ground state, including ^3^MM_(Au:d-sp)_CT and ^3^L_(ph+alkynyl+Tht)_MCT charge transfers, with proportions of 71.34 and 28.66%, respectively.

**Table 1.** Fitting lifetimes of time-resolved PL decays of Au44a in 2-Me-THF under O_2_-purged, N_2_-purged, and ambient conditions.

Condition	τ_1_	τ_2_	τ_av_
Ambient	1.20 μs (51.62%)	9.53 μs (48.38%)	8.54 μs
N_2_-purged	2.26 μs (60.91%)	11.10 μs (39.09%)	8.97 μs
O_2_-purged	1.36 μs (57.39%)	9.47 μs (42.61%)	8.16 μs

To gain further insight into the emissive states, temperature-dependent PH measurements were performed for **Au44a** from 80 to 300 K. As shown in Fig. 5C, a monotonous decrease in the NIR PH intensity and an increase in FWHM can be observed for **Au44a**, indicating more non-radiative relaxation and stronger electron-photon coupling (*46*, *47*), respectively (Fig. 5C). The PH emission of **Au44a** also has an apparent temperature dependence. In contrast to the most gold nanoclusters that demonstrate a slight shift in their maximum emission wavelength response to temperature (*48*–*50*), **Au44a** exhibits a larger redshift of the maximum emission peak from 882 nm for 80 K to 958 nm for 300 K. To be specific, upon heating the sample to 100 K from 80 K, the PH peak position of **Au44a** was constant at 882 nm. Subsequently, an intriguing phenomenon occurred when the temperature was further increased from 100 to 160 K. The maximum PH peak suddenly underwent a notable redshift from 882 to 930 nm. Moreover, a relatively slow redshift occurred as the maximum PH emission shifting from 930 to 958 nm within the temperature range of 160 to 300 K. Similarly, a marked redshift of emission wavelength can also be observed in the temperature-dependent PL spectra of frozen CH_2_Cl_2_ (fig. S25). Why does the increase in temperature cause such a huge change of maximum emission wavelength? This is reminiscent of the reported doped Ru@Au_12_ superatom, which exhibits a large change of maximum emission wavelength because it has a small energy barrier between S_1_ and T_1_ states, enabling the switching between PH and fluorescence (*51*). More recently, a temperature-dependent PL is also observed in the bound ion-pair Ag_29_ + Ag nanoclusters with a peak shift of ~50 nm, due to the excited-state relaxation from the T_1_ state of Ag_29_ cluster to another excited state with triplet character (T_1_′ state) (*52*).

To validate the applicability of the mechanisms elucidated above for the phenomena in **Au44a**, we conducted temperature-dependent time-resolved emission spectrum experiments to investigate the excited state dynamics. As shown in Fig. 5D, a turning point in lifetime can be observed at 100 K. Before this, for example, in the range of 80 to 94 K, only a single-exponential function could fit the decay profile, and a bi-exponential function (τ_1_ and τ_2_) is needed to fit the decay profiles when the turning point is exceeded (Fig. 5E and fig. S26). The contribution of the long-lived component τ_2_ decreased proportionally with declining temperature and ultimately vanished at 80 K, while the short-lived component τ_1_ exhibited an increasing trend and reached unity at 80 K (Fig. 5, F and G). Taking the above results together, we can infer that two triplet excited states (T_1_ and T_1_′) exist in the PH of **Au44a**, which differs from the switching of S_1_ and T_1_ states of Ru@Au_12_ (*51*). Meanwhile, the activation barrier between them is extremely small and can be overcome at temperature over 100 K. Therefore, the relaxation of **Au44a** to its ground state through single- or dual-channel pathways (T_1_ or T_1_ + T_1_′) can be modulated by temperature, rather than the relatively limited modulated approach of introducing of cationic silver(I) complexes in Ag_29_ + Ag nanoclusters (*52*).

**Fig. 6.**
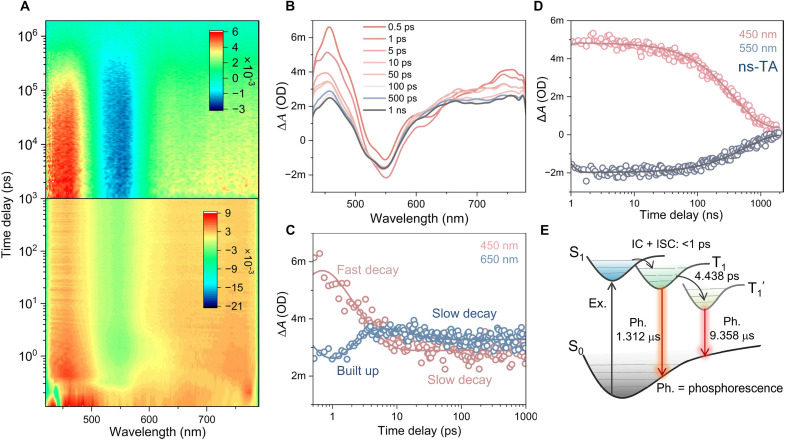
Excited-state dynamics of Au44a. (**A**) Femtosecond– and nanosecond–transient absorption (fs- and ns-TA) data map of **Au44a** in 2-Me-THF excited at 400 nm. (**B**) fs-TA spectra at selected time delays. (**C**) fs-TA kinetic traces and fittings at selected probe wavelengths. (**D**) fs-TA kinetic traces and fittings at selected probe wavelengths. (**E**) Excited-state relaxation diagrams of **Au44a**; the lifetimes of two PH bands obtained from TCSPC. ΔA, change in absorbance; OD, optical density; m, milli.

Moreover, we analyze excited dynamics of **Au44a** via transient absorption (TA) spectroscopy to determine the time evolution of two triplet excited states and corresponding possible transition between them. From the femtosecond/nanosecond (fs/ns)–TA spectra excited by 400-nm laser pulse, a neat ground-state bleach (GSB) signal was observed at the wavelength corresponding to the **Au44a**′s absorption peak α at 550 nm, accompanied by one sharp excited-state absorption (ESA I) signal at approximately 425 to 500 nm, as well as broad ones spanning from 600 nm to the NIR (ESA II) (Fig. 6A). In terms of the time-dependent evolution, within the first picosecond, an ultrafast decay of the ESA I band was observed, while the ESA II remained nearly constant. The decay trends of both ESA I and ESA II within the early time delay may be attributed to the ultrafast intersystem crossing from the singlet excited state to the triplet state (S_n_ → T_1_). As time progresses, ESA I rapidly decays to a flat band within 1 to 10 ps and then slowly decays over 1-ns time window. However, the ESA II gradually builds up over the timescales of 1 to 5.4 ps, illustrating the existence of partial relaxation from ESA I to ESA II (Fig. 6, B and C). This process may be assigned to the conformational relaxation of the excited state {T_1_, which stands for the [MM_(Au)_CT + LM_(Au)_CT] state} to the second most stable triplet state {T_1_′, which stands for the [MM_(Au)_CT + LM_(Au)_CT]′ state}. On the other hand, the excited state lifetimes of the **Au44a** (monitored at the corresponding ESA and GSB signals, Fig. 6D) were found to be comparable with the PL lifetimes measured by time-correlated single-photon counting (TCSPC) (Fig. 5E, bottom). This consistency implies that the two ESA and GSB signals have a similar origin, and the triplet-state emission of the **Au44a**. Figure 6E depicts the relaxation diagram and time constants obtained from fs/ns-TA and TCSPC measurements. These results further corroborate the above deduction that the PH of **Au44a** contains two triplet excited states (T_1_ and T_1_′) with fast picosecond transition time, realizing single/dual-channel NIR PH via thermal control.

## DISCUSSION

In summary, we demonstrate an efficient strategy to synthesize aryl-gold nanoclusters via introducing NaBPh_4_ and NaB(*p*-Ph-F)_4_ as aryl agents that can transfer Ph^−^ and *p*-Ph-F^−^ ligands to protect gold nanoclusters. Two 22-electron arylgold nanoclusters, **Au44a** and **Au44b**, with atomic precise structures have been isolated, each containing six μ_2_-aryl ligands on the surface. The coexistence of two triplet excited states (T_1_ and T_1_′) is responsible for the origin of NIR PH of **Au44a** at room temperature. Meanwhile, the small activation barrier between these two excited states can be overcome at temperature over 100 K, endowing the **Au44a** with thermal-controlled single- or dual-channel PH emission. These results deepen our perception to NIR PL mechanism of gold nanoclusters. Furthermore, this work demonstrates that the aryl groups have potential capability in constructing metal nanoclusters, which opens the door for preparing aryl-stabilized metal nanoclusters using accessible aryl agents.

## MATERIALS AND METHODS

### Synthesis of Au44a

First, [Au(tht)Cl] was synthesized according to the literature method (*53*). Then, [Au(tht)Cl] (6.5 mg, 0.02 mmol) was dissolved in a mixture of 3 ml of CH_2_Cl_2_, and Cl(CH_3_)_2_CC≡CH (5 μl in 1 ml of CH_3_OH), 10 μl of Et_3_N, and NaBPh_4_ (7.5 mg) were added. After stirring for 10 min, a freshly prepared C_4_H_9_NH_2_·BH_3_ solution (0.1 mg in 1 ml of CH_3_OH) was added under vigorous stirring at 0°C with the color of solution changing from pale yellow to dark brown. The reaction continued for 16 hours in the dark. The resultant solution was allowed to evaporate slowly in darkness at 4°C to give black crystals of **Au44a** after 2 days with a yield of ~15% (based on Au).

### Synthesis of Au44b

The synthetic procedure of **Au44b** was similar to that of **Au44a**, except that the NaB(*p*-Ph-F)_4_ was used instead of NaBPh_4_.

### Characterization of Au_44_ nanoclusters

Mass spectra were recorded on a Bruker impact II high-definition mass spectrometer, quadrupole and time-of-flight modules both in the positive ion modes. The data analyses of mass spectra were performed on the basis of the isotope distribution patterns using Compass Data Analysis software (version 4.4). UV-vis absorption spectra were recorded on a Thermo Scientific Evolution 220 UV-vis spectrophotometer. Fourier transform infrared spectra were recorded on a Bruker Tensor II spectrophotometer (Bruker Optics GmbH, Ettlingen, Germany) using a single attenuated total reflectance accessory covering a wave number range from 400 to 4000 cm^−1^. The final spectrum was the average of 32 scans accumulated using Bruker’s Opus software 8.1, taken at a resolution of 4 cm^−1^. The samples were measured under the same mechanical force pushing the samples in contact with the diamond window. PXRD analyses were carried out on a microcrystalline powder using a Rigaku Oxford Diffraction XtaLAB Synergy-S diffractometer using Cu radiation (λ = 1.54184 Å). Morphology of the sample and elemental composition analyses were measured using an SU-8010 field-emission scanning electron microscope (Hitachi Ltd., Tokyo, Japan) equipped with an Oxford-Horiba Inca XMax50 energy-dispersive x-ray spectroscopy attachment (Oxford Instruments Analytical, High Wycombe, England).

### Steady-state PL

The NIR PL, PLE spectra, and quantum yield were recorded on an Edinburgh spectrofluorimeter (FLS920) using a time-correlated single-photon counting technique.

### TA analyses

Samples were prepared by dissolving **Au44a** in 2-Me-THF and transferred to a 2-mm–path length quartz cuvette. An Astrella Ti:Sapphire laser system from Coherent was used as a light source, which operates at a 1-kHz repetition rate, generating 70-fs pulses at 800 nm. The ~70-fs pump laser pulse was generated by a regenerative amplifier system and the optical parametric amplifier (Coherent, Solo). A small portion of the laser fundamental was focused into a sapphire plate to produce a supercontinuum in the visible range, which overlapped with the pump in time and space. Multiwavelength transient spectra were recorded at different pump-probe delay times (Helios Fire, Ultrafast Systems). Time zero, solvent response, and chirp corrections were used using software supplied by the Ultrafast Systems. ns-TA measurements were conducted using the same ultrafast pump pulses along with an electronically delayed supercontinuum light source with a subnanosecond-pulse duration (Nano100, TimeTech Spectra). Details of the x-ray crystallographic analysis and DFT calculations are provided in the Supplementary Materials.

## Supplementary Materials

### This PDF file includes:

Supplementary Materials and Methods Figs. S1 to S26 Tables S1 to S4 References
