# Supplementary material for: Arylgold nanoclusters: Phenyl-stabilized Au44 with thermal-controlled NIR single/dual-channel phosphorescence
Source: Sci Adv. 2024 Feb 14;10(7):eadm6928. doi: 10.1126/sciadv.adm6928 (PMC10866543; doi:10.1126/sciadv.adm6928)
Supplement: Supplementary file 1 — Supplementary Materials and Methods Figs. S1 to S26 Tables S1 to S4 References [file sciadv.adm6928_sm.pdf]

Science Advances
AAAS

Supplementary Materials for

Arylgold nanoclusters: Phenyl-stabilized Au_{44} with thermal-controlled NIR single/dual-channel phosphorescence

Wei-Dan Si *et al.*

Corresponding author: Di Sun, dsun@sdu.edu.cn

Sci. Adv. **10**, eadm6928 (2024)
DOI: 10.1126/sciadv.adm6928

This PDF file includes:

Supplementary Materials and Methods
Figs. S1 to S26
Tables S1 to S4
References

Supplementary Text

X-ray Crystallography

Single crystals of **Au44a**, **Au44b** and **Au22** with appropriate dimensions were chosen under an optical microscope and quickly coated with high vacuum grease (Dow Corning Corporation) to prevent decomposition. The intensity data and cell parameters of **Au44a**, **Au44b** and **Au22** were recorded at 100 K on Bruker D8 VENTURE diffractometer with an Incoatec IμS 3.0 Cu EF microfocus source (55W, Cu Kα, λ = 1.54178 Å) equipped with a PHOTON III C28 detector and an Oxford Cryosystems CryostreamPlus 800 open-fow N_2 cooling device. The raw frame data were processed using SAINT and SADABS to yield the reflection data file (*54*).

These structures were solved using the charge-flipping algorithm, as implemented in the program *SUPERFLIP* (*55*) and refined by full-matrix least-squares techniques against F_o^2 using the SHELXL program (*56*) through the OLEX2 interface (*57*). Hydrogen atoms at carbon were placed in calculated positions and refined isotropically by using a riding model. Appropriate restraints or constraints were applied to the geometry and the atomic displacement parameters of the atoms in the cluster. All structures were examined using the Addsym subroutine of PLATON (*58*) to ensure that no additional symmetry could be applied to the models. Additional solvent molecules, too disordered to be located in the electron density, were taken into account with the SQUEEZE/PLATON procedure (*59*). The unit cell of **Au44b** was found to contain 582 electrons (Z = 4), which are contributed by three CH_2Cl_2 and one H_2O. Pertinent crystallographic data collection and refinement parameters are collated in Table S1. Selected bond lengths are collated in table S2 and S3.

Computational Studies

DFT calculations were performed with the Gaussian 16 suite of programs (*60*). For the optimizations of the **Au44a** and **Au44b**, the gradient-corrected B3PW91 exchange correlation functional, based on the generalized gradient approximation (GGA) was utilized; LANL2DZ(f) (Los Alamos effective core potential double-ζ) basis set was employed for Au atoms, which includes an effective core potential (ECP) for all atoms except those of the first row. The ECP used was proposed by Hay and Wadt, which incorporates the mass velocity and Darwin relativistic effects. The basis set was augmented with *f*-type polarization functions, which is considered to be suitable for this system studied in this work. The 6-31G(d) basis set was used for C, H, O, and S atoms (*61*). The B3PW91 functional is expected to be more suitable for the second and third row transition metal systems (*62*). The LANL2DZ basis set containing relativistic effects has been shown to predict accurately the structure of Au nanoclusters (*63*). Spin-restricted calculations were used for geometry optimization. Harmonic frequencies were then calculated to characterize the stationary points as equilibrium structures with all real frequencies, and to evaluate zero-point energy (ZPE) corrections. The TD-DFT calculations were performed to get the most probable transitions and the orbitals corresponding to the main peaks in the calculated electronic spectrum. A total of 750 and 550 singlet states were chosen in the calculations of **Au44a** and **Au44b**, respectively. The root is set as 1 in the TD-DFT calculations. Data for orbital composition analysis with Mulliken partition are from Gaussian 16 calculations and

further processed with Multiwfn software (*64*).The most probable transitions were determined based on the oscillator strength values and weights. The optical absorption spectra were convoluted with a Gaussian line shape with a half-width at half-height of 0.20 eV.

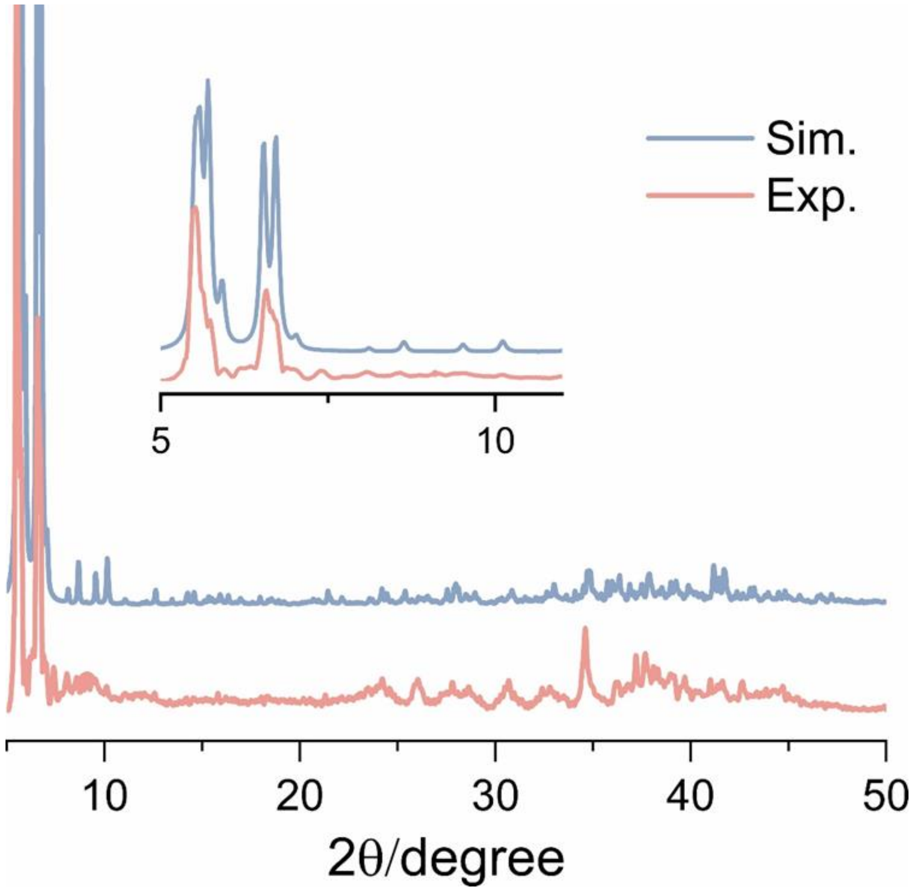

Fig. S1. The PXRD patterns of Au44a.

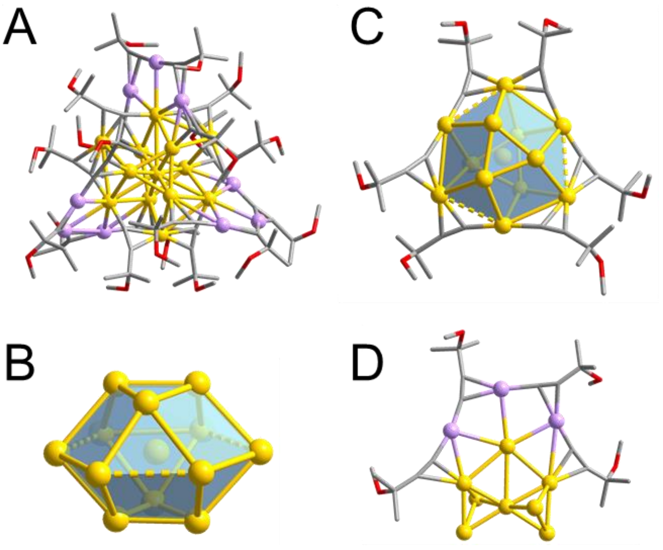

Fig. S2. Structural anatomy of Au22. A) Total structure of Au22. B) Au_{13} kernel. C) The Au_{13} kernel with 6 simplified bridging alkynyl. D) The binding mode of one of the three [$Au_3(CH_3O(CH_3)_2C{\equiv}C)_4$] staple. All hydrogen atoms are omitted for clarity. Color labels: golden and purple, Au; gray, C; red, O.

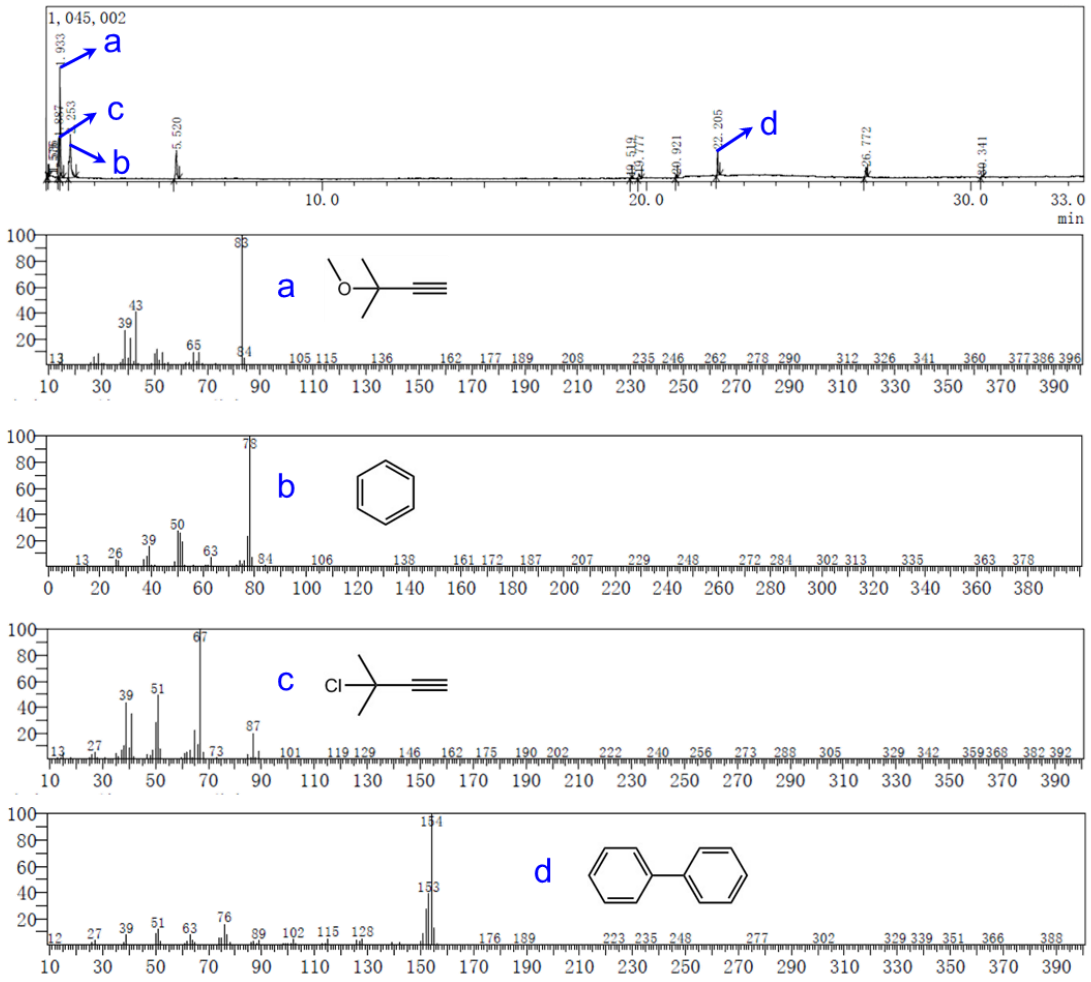

Fig. S3. The GC-MS analysis of the reaction solution of Au44a after 16 h reaction time.

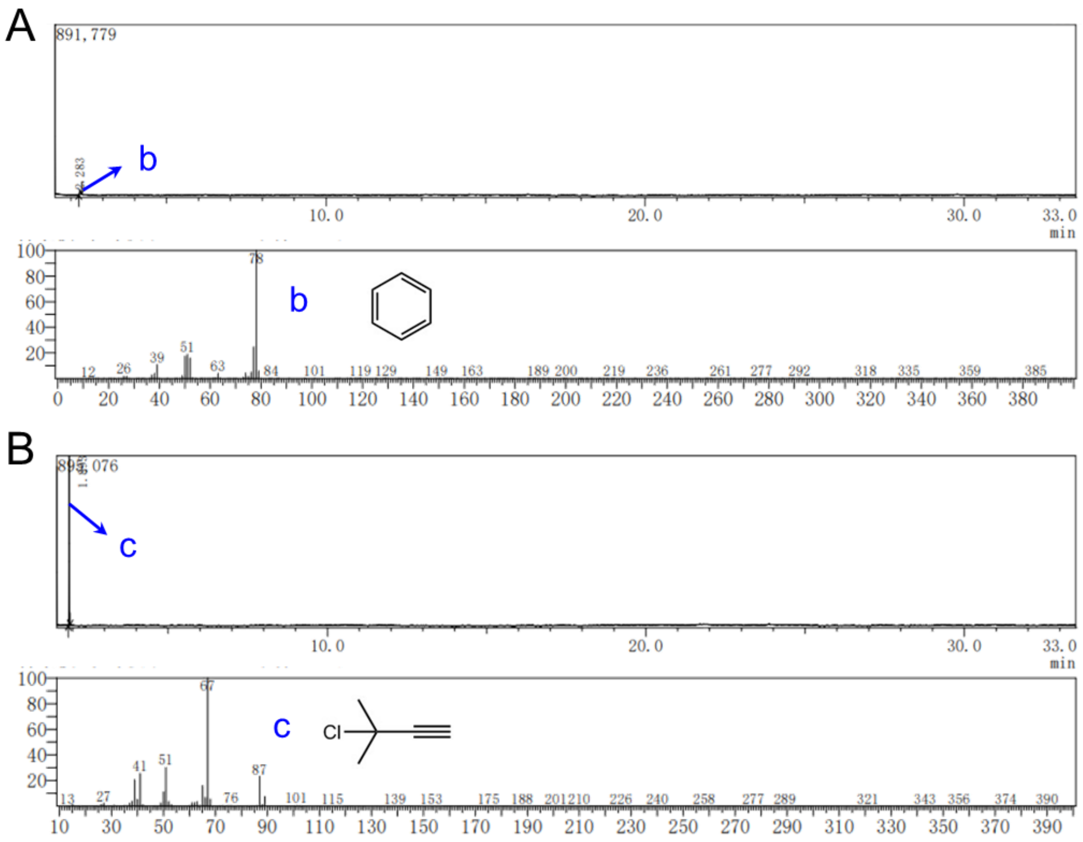

Fig. S4. The GC-MS analysis of the pure $NaBPh_4$ (A) and $Cl(CH_3)_2CC{\equiv}CH$ (B).

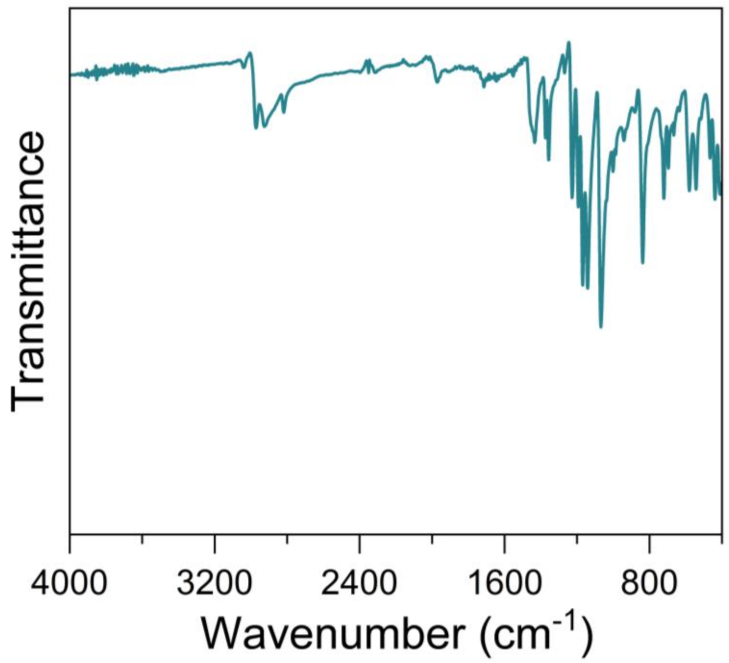

Fig. S5. IR spectrum of Au44a.

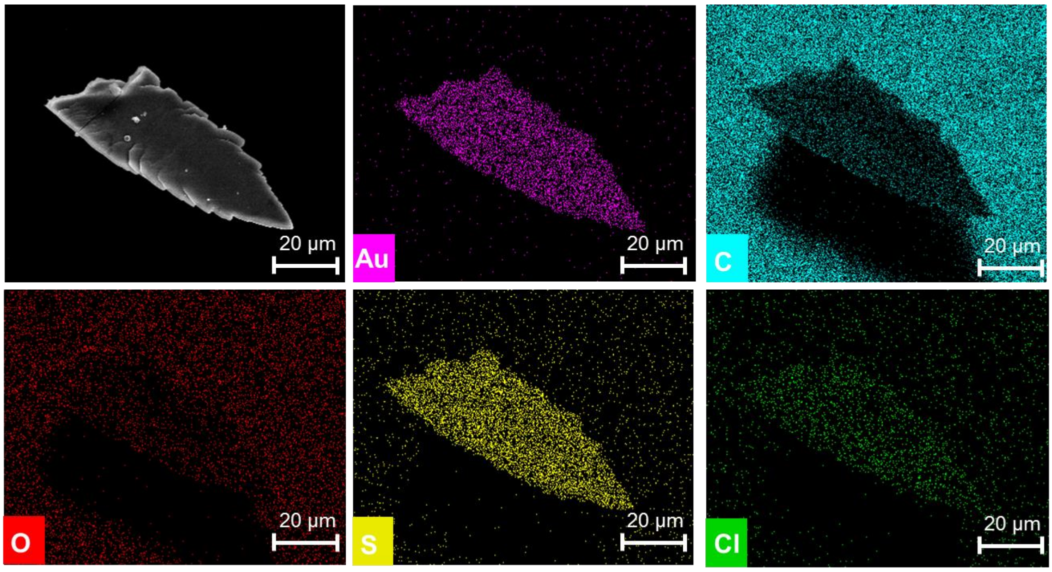

Fig. S6. SEM and elemental mapping images of Au44a.

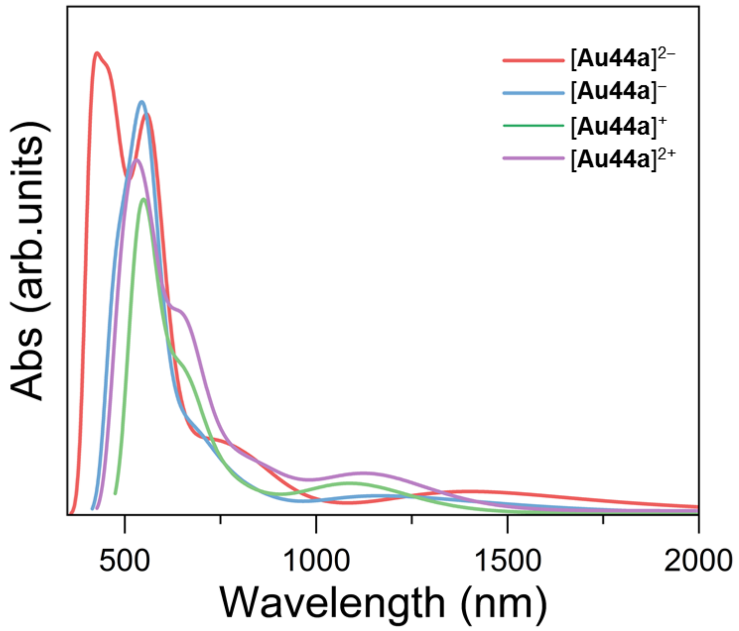

Fig. S7. Calculated absorption spectra of $[Au_{44}(CH_3O(CH_3)_2C{\equiv}C)_{16}(C_6H_5)_6(tht)_2]^q$ (q = -2, -1, 1, 2).

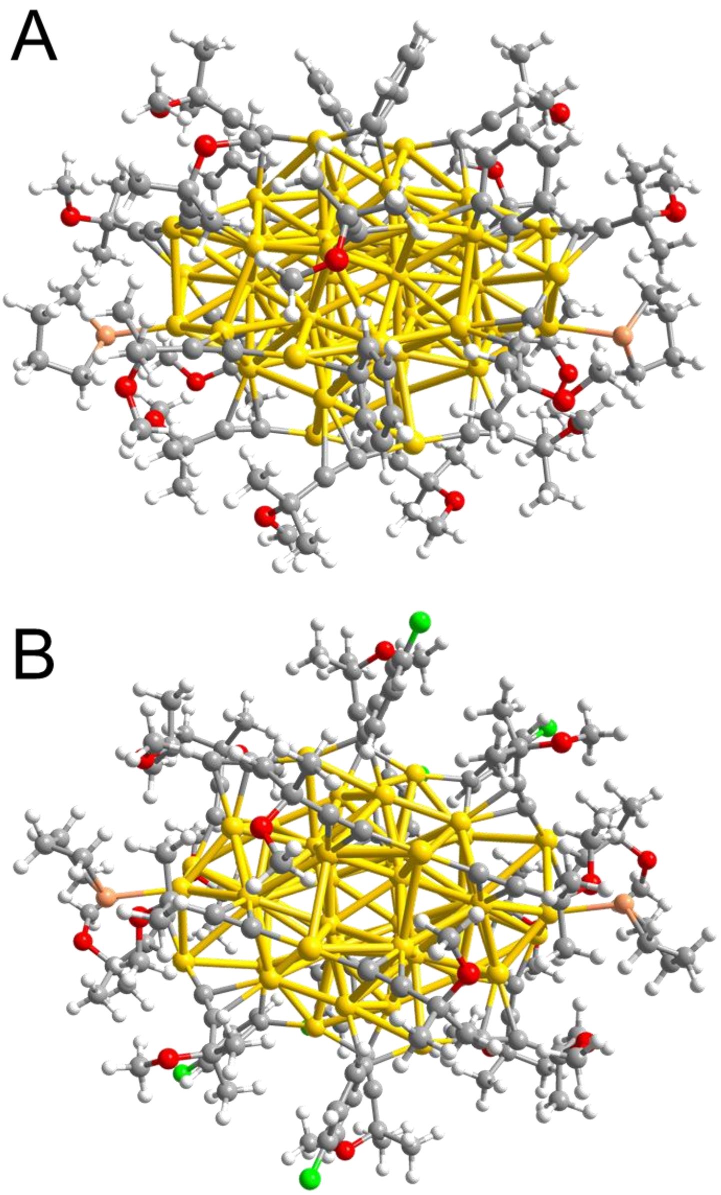

Fig. S8. The optimized DFT structures of Au44a (A) and Au44b (B). Color codes: golden, Au; orange, S; red, O; green, F; gray, C and white, H.

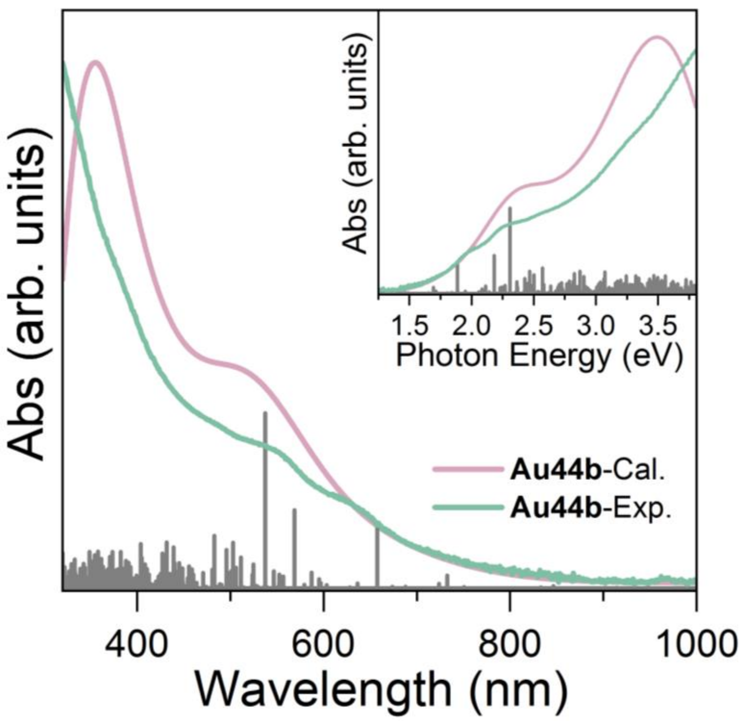

Fig. S9. Experimental (green trace) and calculated (pink trace) absorption spectra of Au44b.

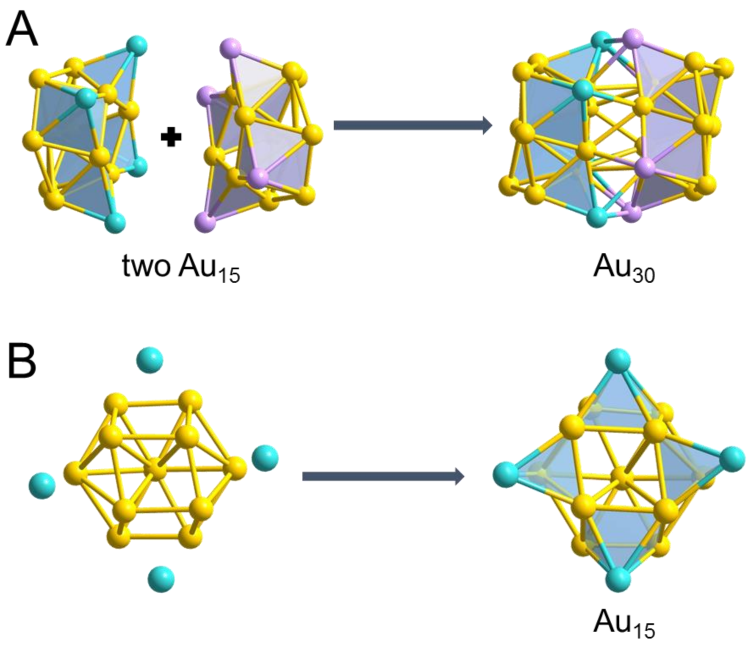

Fig. S10. The Au_{30} (A) and Au_{15} kernels (B) in Au44a.

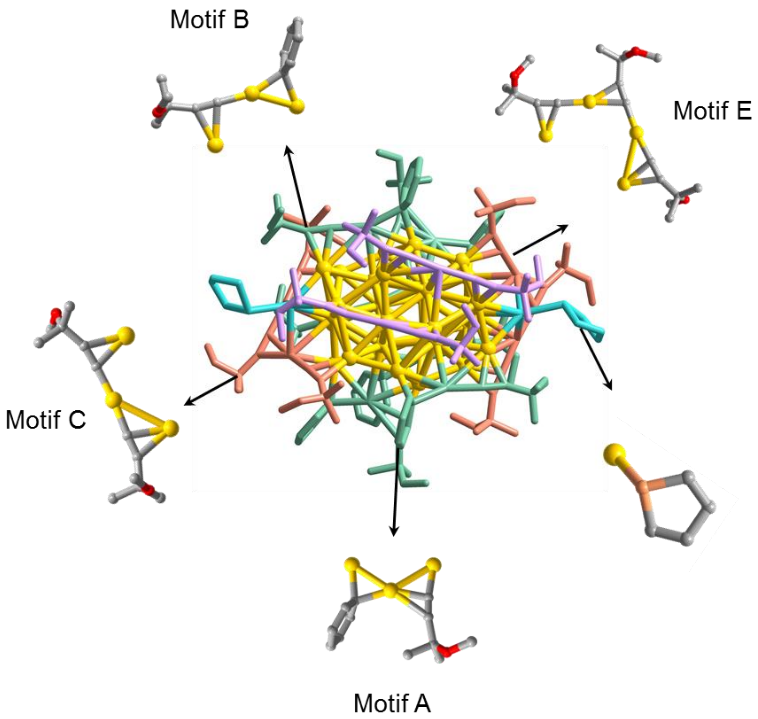

Fig. S11. Binging motifs in Au44a. Green, motif A and motif B; purple, motif C; orange, motif E; blue, Au(tht).

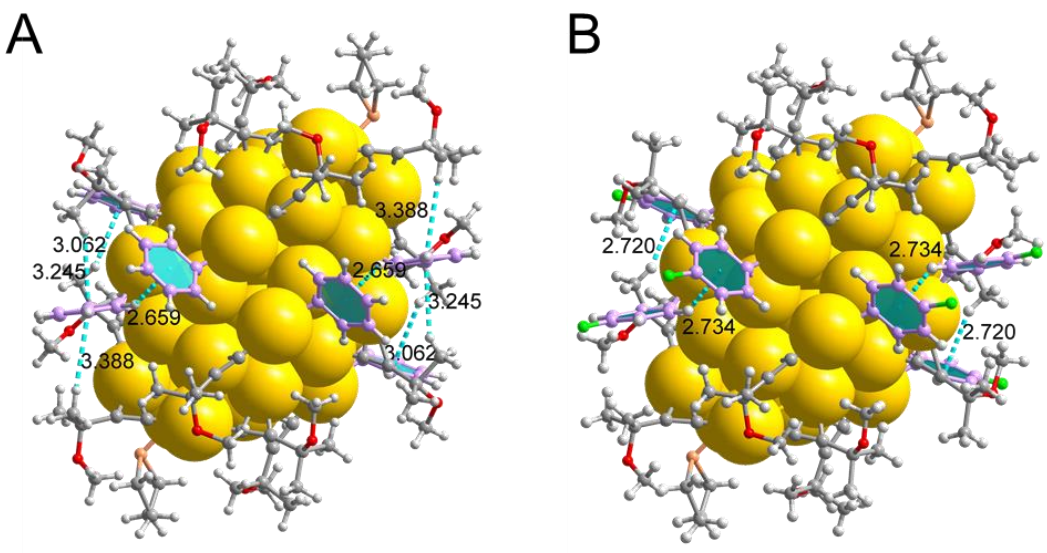

Fig. S12. Intra-cluster interactions (Å) in Au44a (A) and Au44b (B). Color codes: golden, Au; orange, S; red, O; green, F; gray, purple, C and white, H.

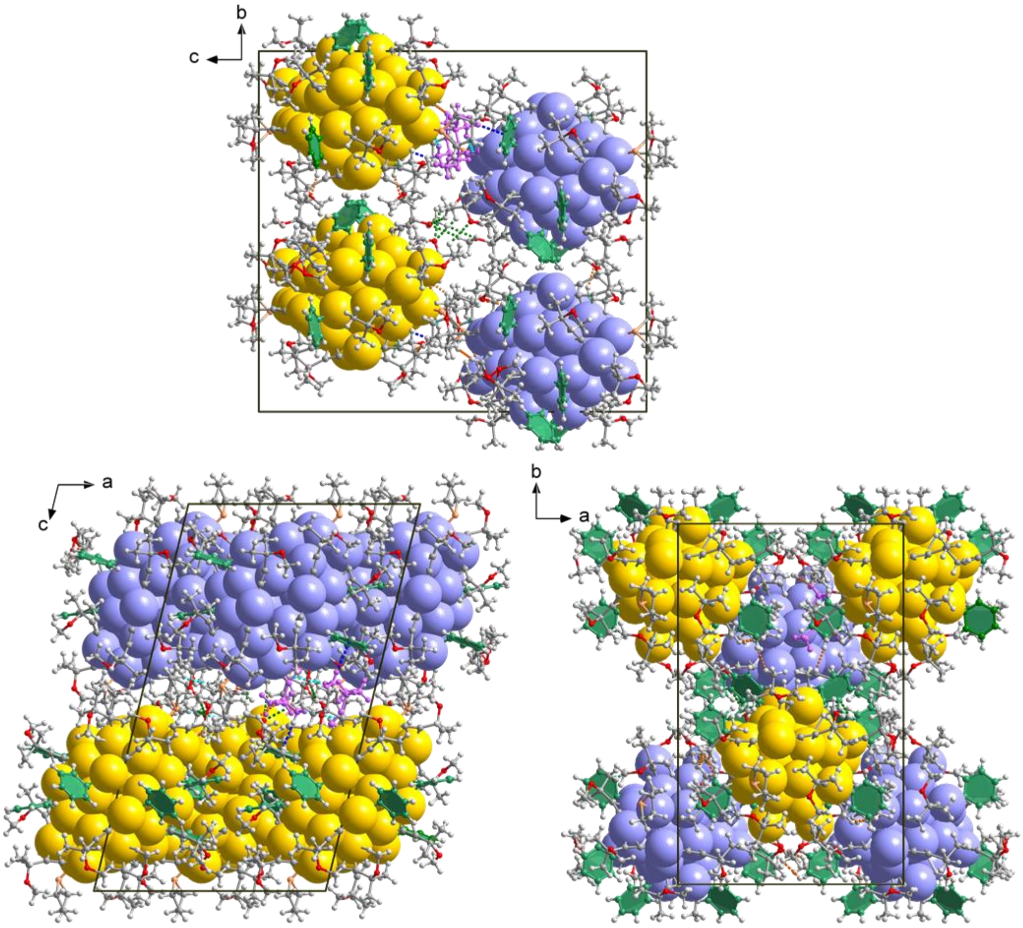

Fig. S13. The self-assembly packing structure of Au44a in space-filling model along the crystallographic *a*, *b* and *c* axis. Color labels of the dashed lines: cyan, $C_{(Me)}$–H···$\pi_{(C\equiv C)}$; blue, $C_{(Me)}$–H···$\pi_{(Ph)}$; orange, H···H; green, C–H···O.

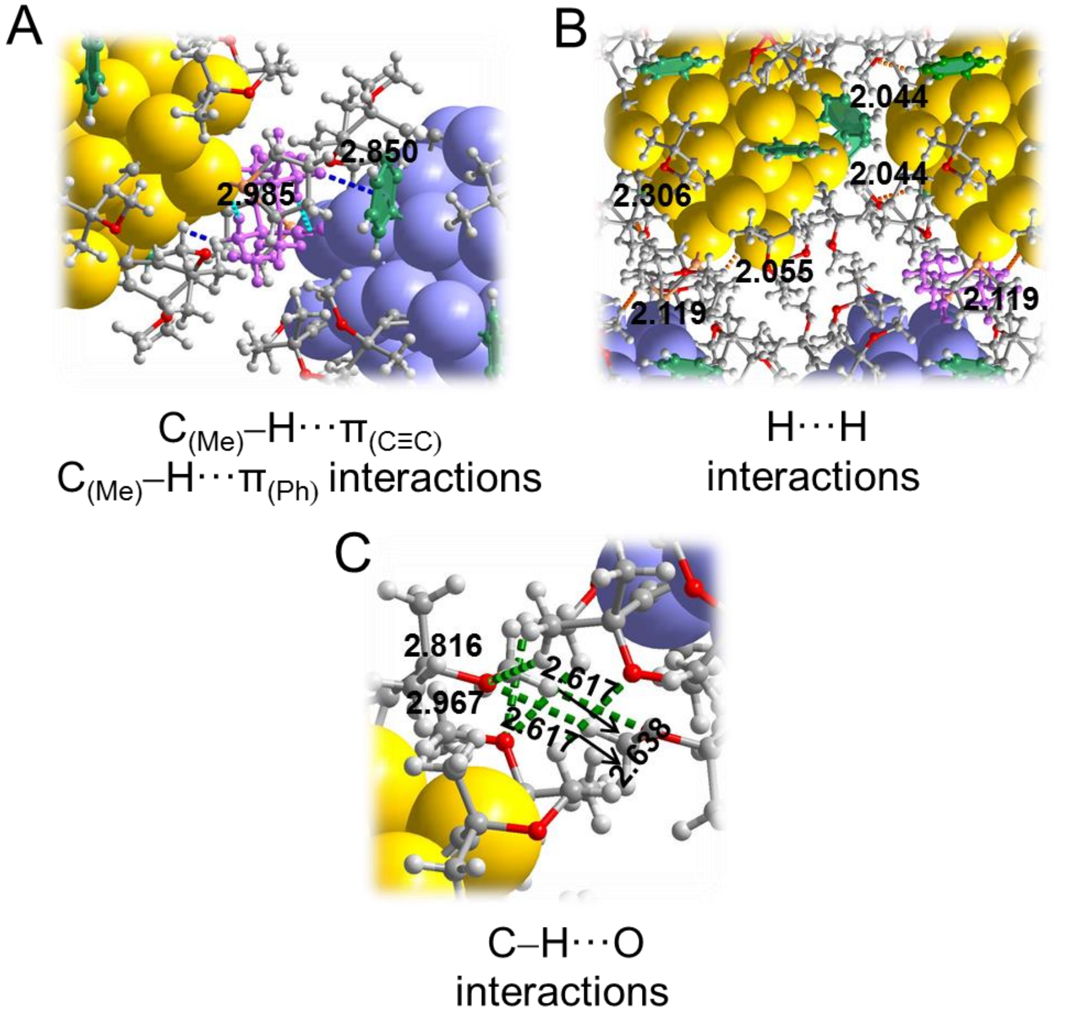

Fig. S14. Inter-cluster interactions (Å) in Au44a. Color labels of the dashed lines: cyan, $C_{(Me)}$–H···$\pi_{(C\equiv C)}$ (a); blue, $C_{(Me)}$–H···$\pi_{(Ph)}$ (a); orange, H···H (b); green, C–H···O (c).

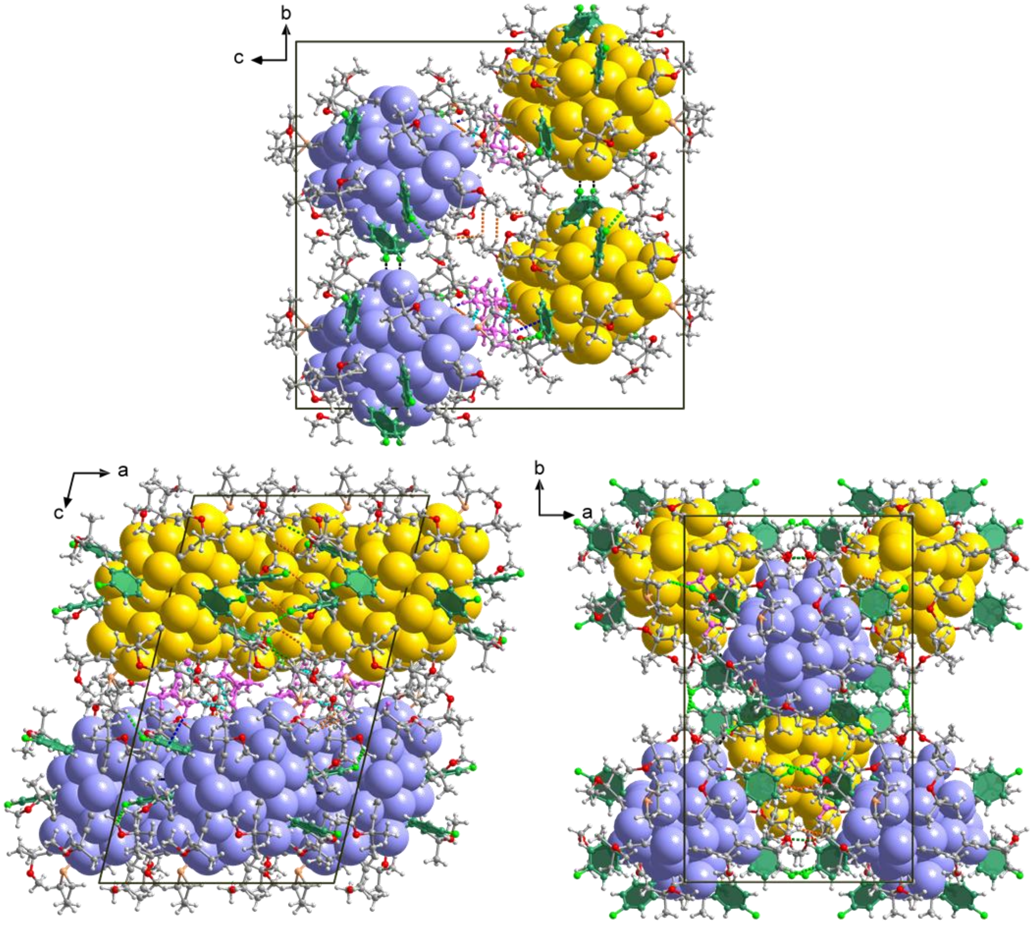

Fig. S15. The self-assembly packing structure of Au44b in space-filling model along the crystallographic *a, b* and *c* axis. Color labels of the dashed lines: cyan, black, C−H···Au; light blue, $C_{(Me)}$−H···$\pi_{(C\equiv C)}$; blue, $C_{(Me)}$−H···$\pi_{(Ph)}$; green, C−H···O; light light, C−H···F; orange, H···H.

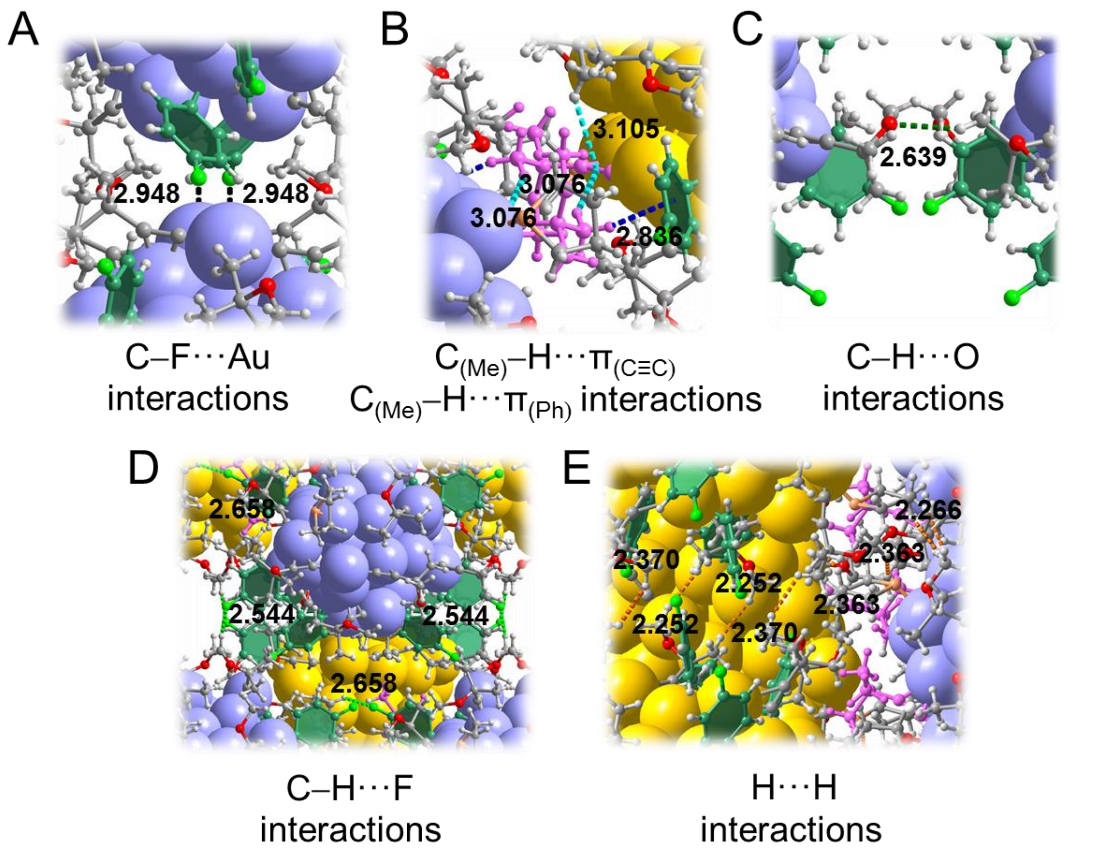

Fig. S16. Inter-cluster interactions (Å) in Au44b. Color labels of the dashed lines: black, C−H···Au (A); light blue, $C_{(Me)}$−H···$\pi_{(C≡C)}$ (B); blue, $C_{(Me)}$−H···$\pi_{(Ph)}$ (B); green, C−H···O (C); light light, C−H···F (D); orange, H···H (E).

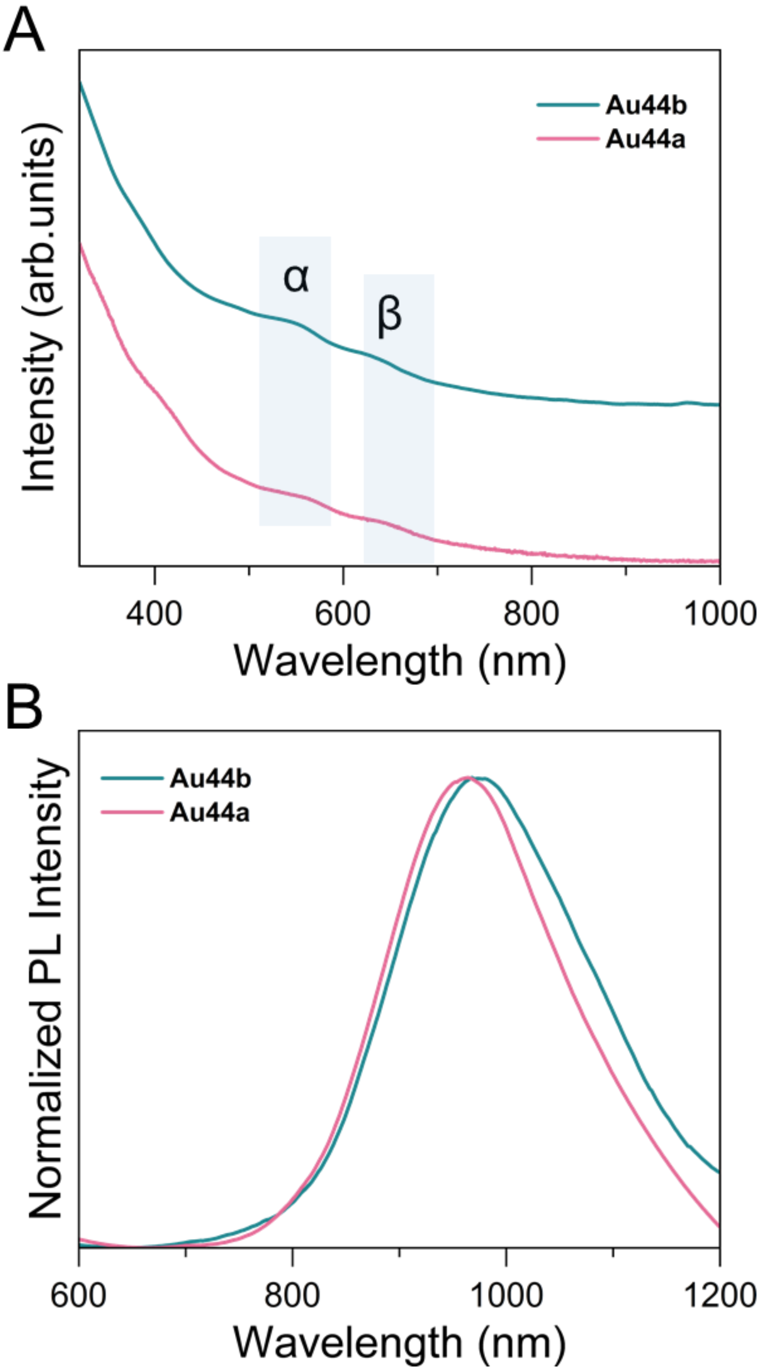

Fig. S17. Comparison of steady-state absorption spectra (A) and NIR emission spectra (B) of Au44a and Au44b in 2-Me-THF under ambient.

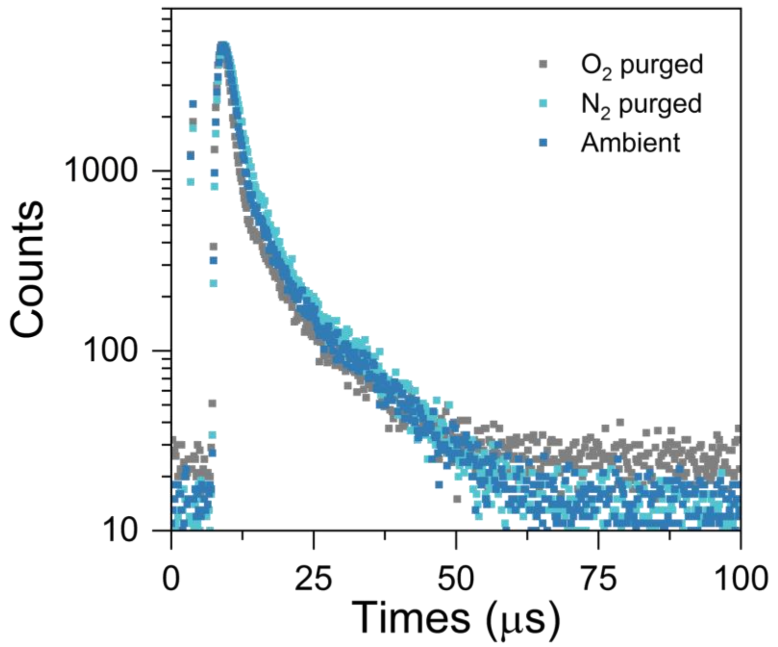

Fig. S18. Time-resolved decay curves of Au44a in solvents under ambient, O_2-purged and N_2-purged conditions.

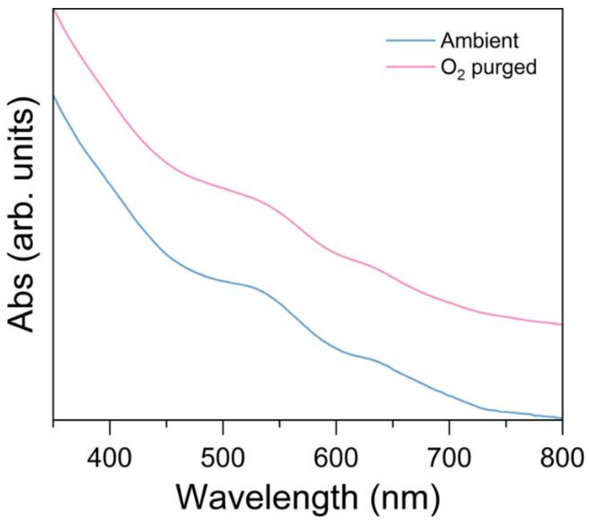

Fig. S19. The absorption spectra of Au44a under ambient and oxygen atmospheres after Xe irradiation.

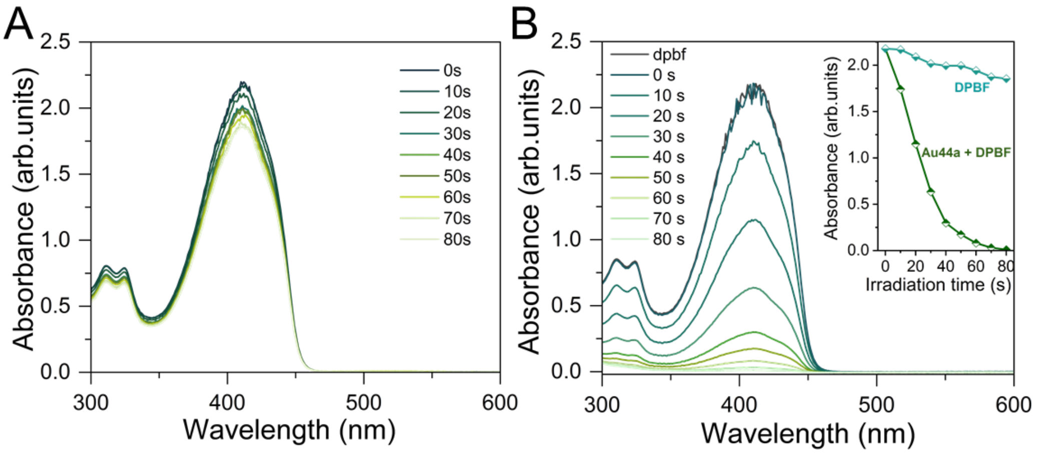

Fig. S20. Detection of singlet oxygen ($^1O_2$) generation. 1, 3-diphenyliso-benzofuran (DPBF) was used to detect the $^1O_2$ generation by **Au44a**. In brief, DPBF in ethanol was prepared, to which the 2-Me-THF solution of **Au44a** was added to give final concentrations of 1 mM for DPBF and 5×10^{-2} μM for **Au44a**. The mixed solution was irradiated by Xe lamp, and the adsorption spectra were recorded on a Thermo Scientific Evolution 220 UV-visible spectrophotometer. Time-dependent absorption spectra of a DPBF-containing solution of **Au44a** (A) and a DPBF solution of EtOH (B) in air. Inset: the absorption of DPBF at 411 nm as a function of irradiation time.

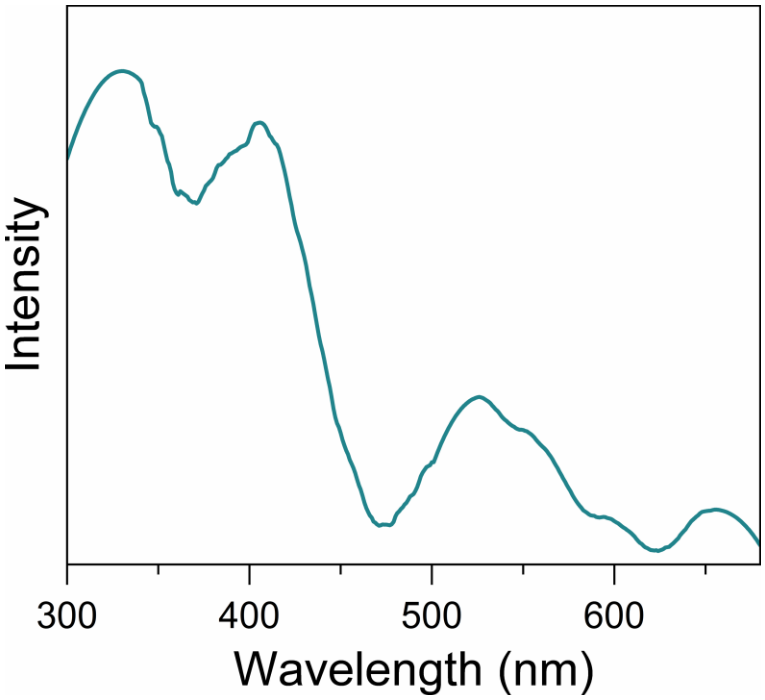

Fig. S21. PLE of Au44a in 2-Me-THF under ambient.

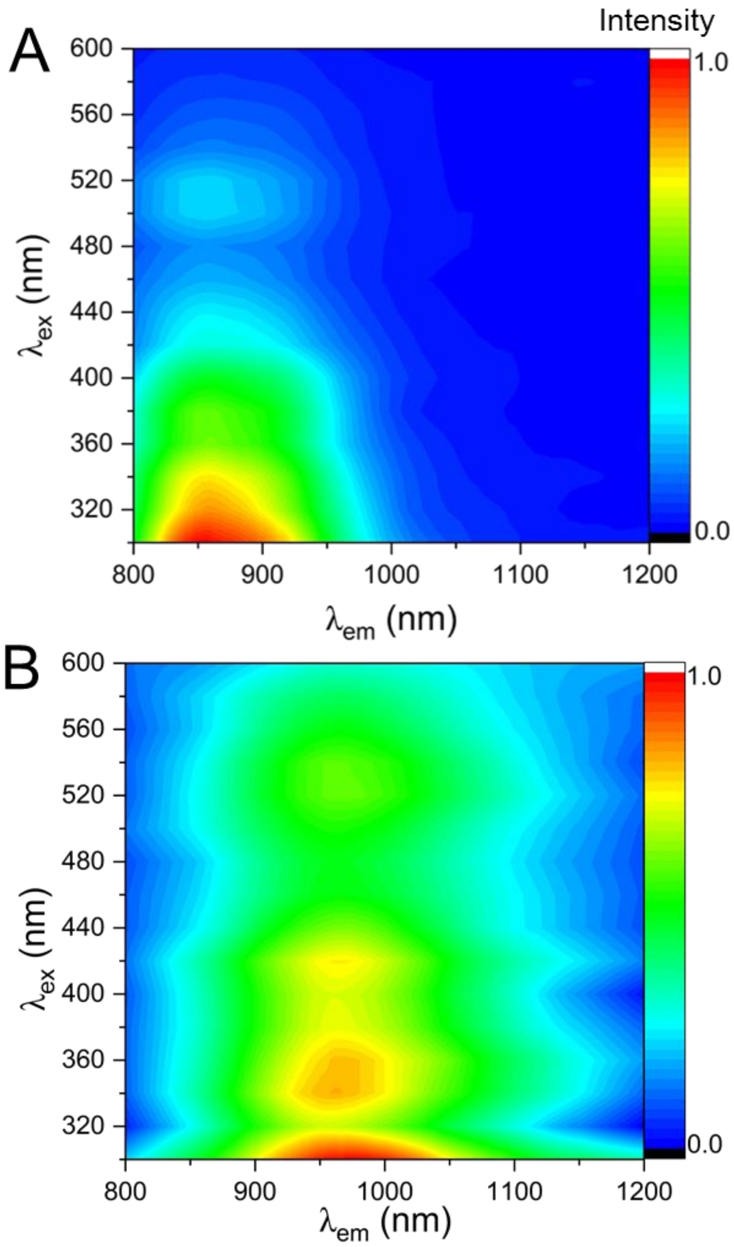

Fig. S22. Excitation-dependent emission mapping of Au44a in 2-Me-THF under 80 K (A) and 300 K (B).

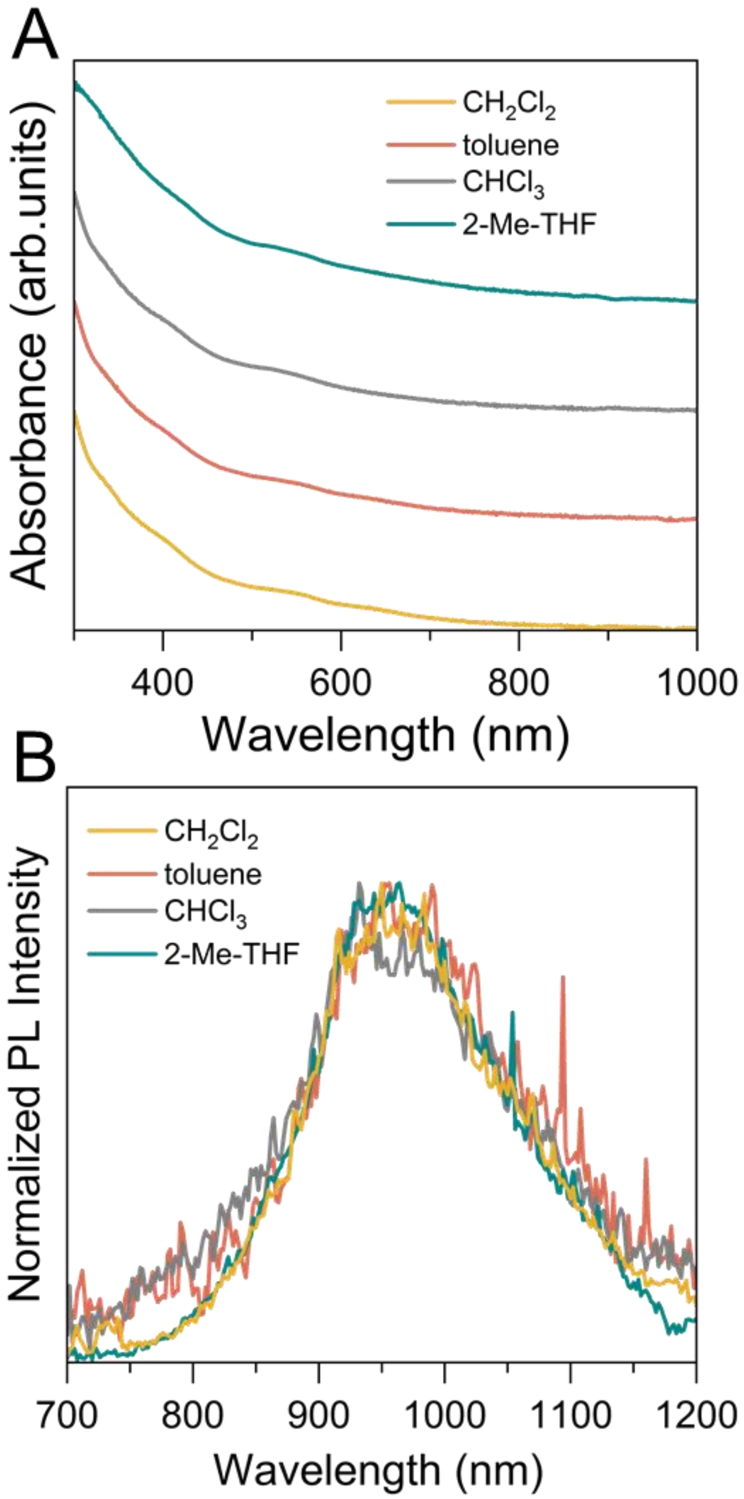

Fig. S23. Comparison of the steady-state absorption spectra (A) and NIR emission spectra (B) of Au44a in four solvents with different polarity.

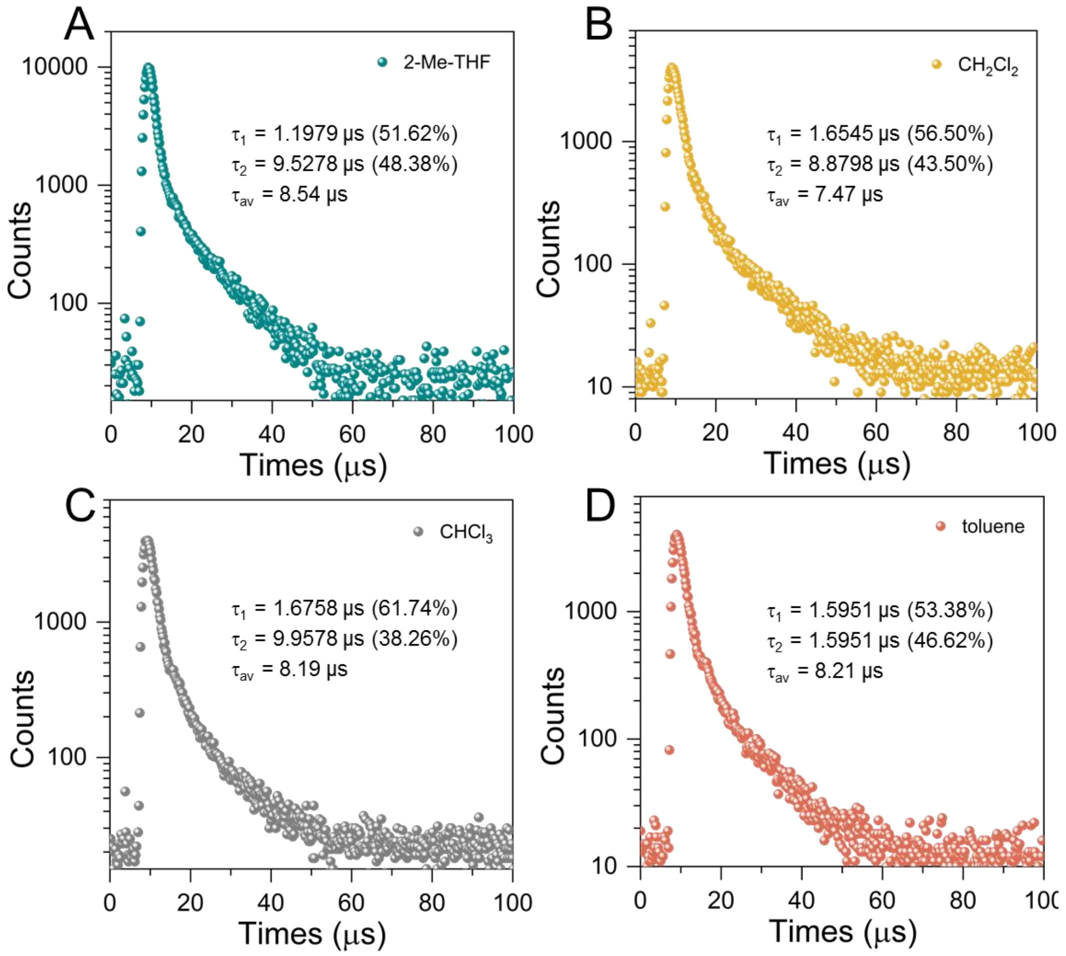

Fig. S24. Time-resolved decay curves of Au44a in four solvents with different polarity.

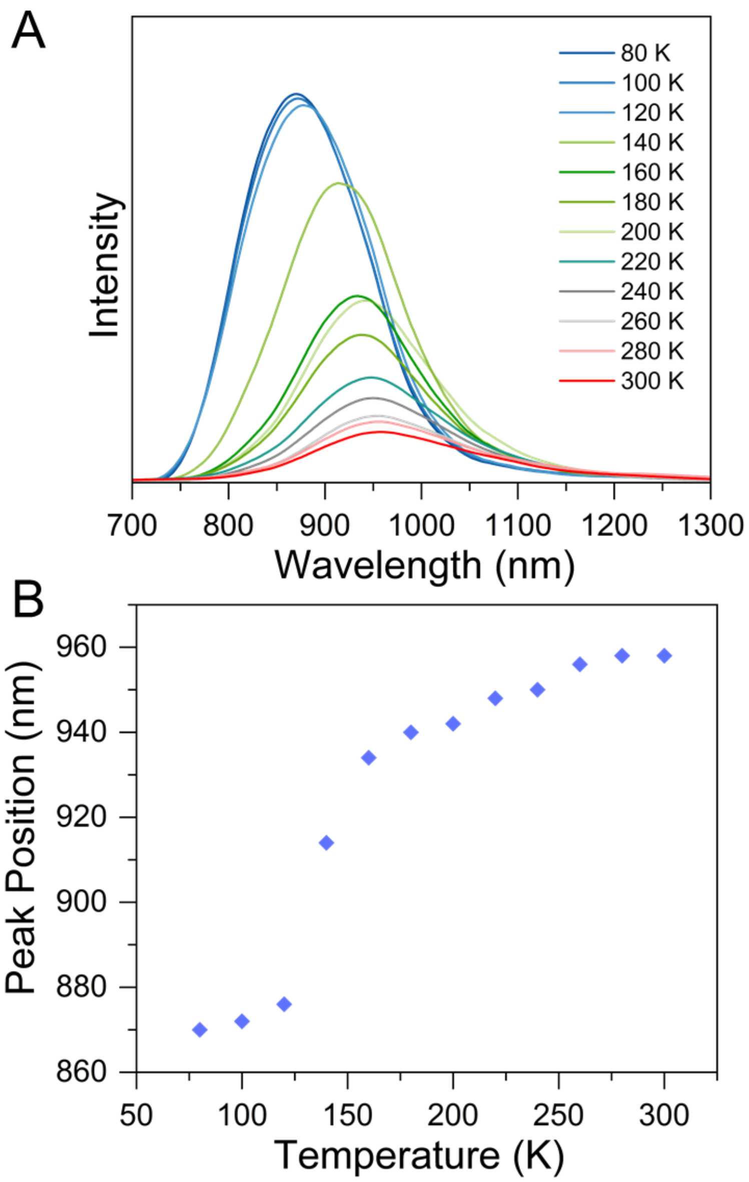

Fig. S25. PL spectra of Au44a in DCM. (A) Temperature-dependent NIR emission spectra of **Au44a** in DCM. (B) The main peak position as a function of temperature.

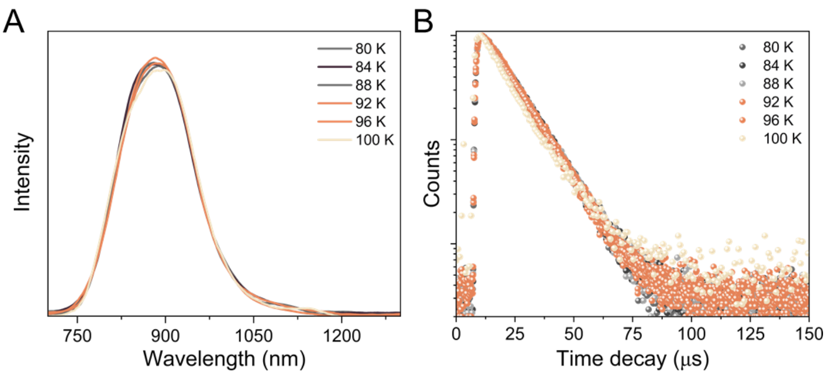

C

Tem. (K)	τ_1 (μs)	x_1	τ_2 (μs)	x_2
80	10.99	100%	0	/
84	11.03	100%	0	/
88	10.90	100%	0	/
92	10.93	100%	0	/
96	10.89	100%	0	/
100	9.539	68.22%	15.69	31.78%

Fig. S26. Temperature-dependent NIR PL within a specific range. (A) Temperature-dependent NIR emission spectra of Au44a in 2-Me-THF (80-100 K). (B) The decay curves of Au44a in the range of 80-100 K. (C) The detail contributions of τ_1 and τ_2 at different temperatures.

Table S1. Crystal data and structure refinement for Au44a and Au44b.

Identification code	**Au44a**	**Au44b**
Empirical formula	$C_{142}H_{194}Au_{44}Cl_4O_{18}S_2$	$C_{140}H_{184}Au_{44}F_6O_{16}S_2$
Formula weight	11061.41	10967.51
Temperature/K	100.0	100.0
Crystal system	monoclinic	monoclinic
Space group	*C*2/*c*	*C*2/*c*
a/Å	19.0064(11)	19.189(18)
b/Å	29.5676(11)	29.91(3)
c/Å	32.6750(13)	32.59(2)
α/°	90	90
β/°	103.746(3)	103.82(3)
γ/°	90	90
Volume/Å^3	17836.6(14)	18166(27)
Z	4	4
ρ_{calc}g/cm^3	4.119	4.010
μ/mm^{-1}	67.207	65.487
F(000)	19064.0	18856.0
2Θ range for data collection/°	5.978 to 134.15	5.658 to 134.158
Index ranges	$-22 \le h \le 22$, $-34 \le k \le 35$, $-39 \le l \le 39$	$-22 \le h \le 22$, $-34 \le k \le 35$, $-38 \le l \le 38$
Radiation, CuKα	1.54184	1.54184
Reflections collected	81893	48557
Independent reflections	15912 [R_{int} = 0.0998, R_{sigma} = 0.0773]	16135 [R_{int} = 0.1090, R_{sigma} = 1255]
Data/restraints/parameters	15912/456/1028	16135/449/921
Goodness-of-fit on F^2	1.090	0.968
Final R indexes [I>=2σ (I)]	R_1 = 0.0589, wR_2 = 0.1525	R_1 = 0.0799, wR_2 = 0.2025
Final R indexes [all data]	R_1 = 0.827, wR_2 = 0.1656	R_1 = 0.1440, wR_2 = 0.2525
Largest diff. peak/hole / e Å^{-3}	3.77/-2.92	3.93/-3.25

Table S2. Au–S and Au–C bond lengths (Å) for Au44a and Au44b.

**Au44a**		**Au44b**	
Au—S		Au—S	
Au2—S1	2.381(6)	Au2—S1	2.373(13)
Au—C(sp)		Au—C(sp)	
Au3—C30	1.97(3)	Au3—C69[1]	1.95(4)
Au3—C39	1.99(3)	Au5—C7	2.05(7)
Au5—C45	1.99(2)	Au8—C1	2.23(4)
Au7—C24	1.98(3)	Au8—C12	2.25(5)
Au7—C37	2.00(3)	Au9—C4	2.27(6)
Au9—C28	1.97(2)	Au9—C31	2.27(6)
Au10—C5	2.27(2)	Au11—C7[1]	2.09(6)
Au10—C17	2.23(2)	Au13—C1	1.92(5)
Au11—C45	2.33(3)	Au13—C37[1]	1.89(5)
Au11—C57	2.29(3)	Au14—C27	2.31(4)
Au13—C21	2.04(3)	Au14—C55	2.16(4)
Au14—C34	2.32(3)	Au15—C18	2.22(6)
Au14—C39	2.28(2)	Au15—C27	1.96(5)
Au16—C37	2.26(2)	Au15—C36	2.29(6)
Au16—C66	2.32(2)	Au18—C10	2.26(5)
Au17—C28	2.23(3)	Au18—C37	2.37(5)
Au17—C43	2.24(3)	Au19—C14	2.10(6)
Au20—C15	2.32(3)	Au19—C18	2.02(5)
Au20—C30	2.28(3)	Au20—C14	2.22(5)
Au21—C21[1]	2.13(5)	Au20—C23	2.35(5)
Au21—C35	2.12(3)	Au21—C31[1]	1.91(6)
Au21—C38[1]	2.58(4)	Au22—C8	2.27(4)
Au22—C5	2.00(3)	Au22—C69	2.19(4)
Au22—C24	2.26(3)		
Au22—C26	2.19(3)		
Au—C(sp^2)		Au—C(sp^2)	
Au5—C44	2.13(3)	Au3—C25	2.18(2)
Au9—C54	2.15(3)	Au5—C54	2.18(4)
Au12—C54	2.14(3)	Au7—C54	2.15(3)
Au19—C44	2.15(3)	Au10—C25	2.16(2)
Au21—C35	2.12(3)	Au17—C41	2.24(4)
Au15—C35	2.15(3)	Au21—C41	2.13(4)
[1]1-X,+Y,3/2-Z		[1]1-X,+Y,1/2-Z	

Table S3. Selected bond lengths (Å) and angle (°) for Au44a and Au44b.

**Au44a**			
Au1—Au2	2.8252(13)	Au6—Au20[1]	3.2656(13)
Au1—Au6[1]	2.9560(12)	Au7—Au11	2.9400(13)
Au1—Au7	3.1423(13)	Au7—Au16	3.3409(12)
Au1—Au8[1]	3.2113(12)	Au8—Au8[1]	2.8760(17)
Au1—Au10	3.0015(13)	Au8—Au11[1]	2.7947(12)
Au1—Au11	2.7463(14)	Au8—Au13[1]	2.8206(13)
Au1—Au15	2.7671(13)	Au8—Au16[1]	2.7597(12)
Au1—Au16	2.9342(13)	Au8—Au18[1]	2.8083(12)
Au1—Au17[1]	2.8089(13)	Au8—Au18	2.7723(13)
Au1—Au18[1]	3.0450(12)	Au8—Au19	2.8089(13)
Au1—Au20	2.7736(14)	Au8—Au19[1]	2.8668(12)
Au1—Au22	3.1450(12)	Au9—Au12	2.7297(12)
Au2—Au7	3.2539(15)	Au9—Au13[1]	3.2866(15)
Au2—Au17[1]	2.6823(13)	Au9—Au17	3.2823(12)
Au2—Au20	2.7862(13)	Au10—Au12	2.7213(12)
Au2—Au22	2.9308(14)	Au10—Au15	3.0226(14)
Au3—Au4[1]	2.9564(14)	Au10—Au18[1]	2.7217(12)
Au3—Au14	3.2864(13)	Au10—Au20	3.0130(13)
Au3—Au14[1]	3.3438(14)	Au11—Au15	2.6983(14)
Au4—Au4[1]	2.7816(16)	Au11—Au16	2.9304(13)
Au4—Au6[1]	3.1497(12)	Au11—Au19[1]	2.9358(13)
Au4—Au6	2.8453(13)	Au12—Au13[1]	3.0260(14)
Au4—Au10[1]	2.8097(12)	Au12—Au14	3.2542(13)
Au4—Au12[1]	2.7358(13)	Au12—Au18[1]	2.8373(13)
Au4—Au14[1]	2.8744(12)	Au13—Au16	2.6777(12)
Au4—Au14	2.7170(13)	Au13—Au18	2.8815(13)
Au4—Au18	2.6868(13)	Au13—Au21[1]	2.9104(13)
Au4—Au20[1]	2.8111(12)	Au14—Au20[1]	3.0463(13)
Au5—Au8[1]	3.0155(14)	Au15—Au18[1]	2.8432(12)
Au5—Au19	2.6969(13)	Au15—Au19[1]	2.9017(13)
Au6—Au8	2.7474(13)	Au15—Au21	2.6999(14)
Au6—Au9	2.9998(13)	Au15—Au22	2.8988(13)
Au6—Au12	2.8922(12)	Au16—Au17[1]	2.9142(14)
Au6—Au13[1]	2.8309(13)	Au17—Au20[1]	2.6816(13)
Au6—Au14	2.6887(14)	Au18—Au19	2.7178(14)
Au6—Au16[1]	2.8910(13)	Au18—Au21[1]	3.2066(14)
Au6—Au17	2.8527(12)	Au19—Au21[1]	3.1227(15)
Au6—Au18[1]	3.0954(12)		
[1]1-X,+Y,3/2-Z			

Au44b			
Au1—Au3[1]	3.009(3)	Au6—Au16[1]	2.821(3)
Au1—Au4	2.847(3)	Au6—Au16	2.779(3)
Au1—Au4[1]	3.177(3)	Au6—Au17	2.714(3)
Au1—Au6[1]	3.085(3)	Au7—Au9	2.703(3)
Au1—Au8	2.674(3)	Au7—Au12	2.755(3)
Au1—Au10[1]	2.897(3)	Au7—Au14	2.992(3)
Au1—Au11	2.818(3)	Au7—Au15	2.901(3)
Au1—Au12	2.953(3)	Au7—Au17	2.928(3)
Au1—Au16	2.741(3)	Au8—Au10[1]	3.217(3)
Au1—Au18	3.268(3)	Au8—Au13	3.286(3)
Au1—Au20	2.888(3)	Au8—Au13[1]	3.340(3)
Au1—Au22	2.858(3)	Au8—Au18	3.055(3)
Au2—Au12	2.815(3)	Au9—Au12	2.749(3)
Au2—Au15	2.931(3)	Au9—Au16	2.800(3)
Au2—Au18	2.785(3)	Au9—Au17	2.931(3)
Au2—Au19	3.229(4)	Au9—Au19	2.942(3)
Au2—Au22	2.686(3)	Au9—Au20	2.949(3)
Au3—Au8[1]	3.377(3)	Au10—Au11[1]	3.050(3)
Au3—Au10	2.746(3)	Au10—Au14	2.721(3)
Au3—Au11[1]	3.350(3)	Au11—Au16	2.817(3)
Au3—Au22[1]	3.290(3)	Au11—Au20	2.686(3)
Au4—Au4[1]	2.782(4)	Au12—Au14	3.017(3)
Au4—Au6	2.674(3)	Au12—Au15	3.169(3)
Au4—Au8[1]	2.891(3)	Au12—Au16	3.211(3)
Au4—Au8	2.719(3)	Au12—Au18	2.760(3)
Au4—Au10	2.735(3)	Au12—Au19	3.132(3)
Au4—Au13[1]	2.912(3)	Au12—Au20	2.925(3)
Au4—Au14	2.812(3)	Au12—Au22	2.802(3)
Au4—Au18	2.814(3)	Au14—Au18	3.007(3)
Au5—Au6	3.190(3)	Au16—Au16[1]	2.896(4)
Au5—Au7	2.730(3)	Au16—Au17[1]	2.851(3)
Au5—Au11[1]	2.854(3)	Au16—Au17	2.795(3)
Au5—Au17	3.137(4)	Au16—Au20	2.758(3)
Au6—Au7	2.831(3)	Au16—Au21[1]	3.022(3)
Au6—Au10	2.825(3)	Au17—Au21	2.726(3)
Au6—Au11[1]	2.892(3)	Au18—Au22	2.678(3)
Au6—Au12	3.058(3)	Au19—Au20	3.360(3)
Au6—Au14	2.742(3)	Au20—Au22	2.905(3)
[1]1-X,+Y,1/2-Z			

Table S4. Bond angle (°) for Au44a and Au44b.

Au44a		Au44b	
Au22—C5—Au10	110.3(11)	Au13—C1—Au8	104(2)
Au13—C21—Au21[1]	88.3(16)	Au10—C25—Au3	78.3(5)
Au7—C24—Au22	111.2(12)	Au5—C7—Au11[1]	87(2)
Au9—C28—Au17	102.6(10)	Au19—C14—Au20	102(2)
Au3—C30—Au20	110.3(12)	Au19—C18—Au15	111(3)
Au21—C35—Au15	78.3(9)	Au21—C41—Au17	77.2(8)
Au7—C37—Au16	103.2(11)	Au15—C27—Au14	109.6(19)
Au3—C39—Au14	100.5(11)	Au21[1]—C31—Au9	112(3)
Au5—C44—Au19	78.1(10)	Au13[1]—C37—Au18	109(2)
Au5—C45—Au11	106.1(11)	Au7—C54—Au5	78.3(8)
Au12—C54—Au9	79.0(9)	Au3[1]—C69—Au22	104.9(17)
[1]1-X,+Y,3/2-Z		[1]1-X,+Y,1/2-Z	

REFERENCES AND NOTES

1. R. P. Herrera, M. C. Gimeno, Main avenues in gold coordination chemistry. *Chem. Rev.* **121**, 8311–8363 (2021).

2. M.-C. Tang, M.-Y. Leung, S.-L. Lai, M. Ng, M.-Y. Chan, V. W.-W. Yam, Realization of thermally stimulated delayed phosphorescence in arylgold(III) complexes and efficient gold(III) based blue-emitting organic light-emitting devices. *J. Am. Chem. Soc.* **140**, 13115–13124 (2018).

3. L. Rocchigiani, M. Bochmann, Recent advances in gold(III) chemistry: Structure, bonding, reactivity, and role in homogeneous catalysis. *Chem. Rev.* **121**, 8364–8451 (2021).

4. Q. H. Wei, L. Y. Zhang, G. Q. Yin, L. X. Shi, Z. N. Chen, Luminescent heteronuclear $Au^{I}_{5}Ag^{I}_{8}$ complexes of $\{1,2,3\text{-}C6(C6H4R\text{-}4)3\}^{3-}$ (R = H, CH_3, Bu^{t}) by cyclotrimerization of arylacetylides. *J. Am. Chem. Soc.* **126**, 9940–9941 (2004).

5. R. Uson, A. Laguna, Recent Development in arylgold chemistry. *Coord. Chem. Rev.* **70**, 1–50 (1986).

6. E. J. Fernandez, A. Laguna, M. E. Olmos, Recent development in arylgold chemistry. *Adv. Organomet. Chem.* **52**, 77–141 (2005).

7. A. Maity, A. N. Sulicz, N. Deligonul, M. Zeller, A. D. Hunter, T. G. Gray, Suzuki−Miyaura coupling of arylboronic acids to gold(III). *Chem. Sci.* **6**, 981–986 (2015).

8. A. Sladek, S. Hofreiter, M. Paul, H. Schmidbaur, Sodium tetraphenylborate as a phenylating agent for gold(I) complexes. *J. Organomet. Chem.* **501**, 47–51 (1995).

9. Y. Tsuchido, R. Abe, T. Ide, K. Osakada, A macrocyclic gold(I)-biphenylene complex: Triangular molecular structure with twisted Au_2(diphosphine) corners and reductive elimination of [6]cycloparaphenylene. *Angew. Chem. Int. Ed.* **59**, 22928–22932 (2020).

10. A. Laguna, M. Laguna, M. C. Gimeno, P. G. Jones, Synthesis and X-ray characterization of the neutral organometallic gold cluster $[Au_{10}(C_6F_5)_4(PPh_3)_5]$. *Organometallics* **11**, 2759–2760 (1992).

11. E. S. Smirnova, A. M. Echavarren, A hexanuclear gold cluster supported by three-center-two-electron bonds and aurophilic interactions. *Angew. Chem. Int. Ed.* **52**, 9023–9026 (2013).

12. R. H. Adnan, J. M. L. Madridejos, A. S. Alotabi, G. F. Metha, G. G. Andersson, A review of state of the art in phosphine ligated gold clusters and application in catalysis. *Adv. Sci.* **9**, 2105692 (2022).

13. X. Kang, M. Zhu, Tailoring the photoluminescence of atomically precise nanoclusters. *Chem. Soc. Rev.* **48**, 2422–2457 (2019).

14. Z. H. Liu, Z. N. Wu, Q. F. Yao, Y. T. Cao, O. J. H. Chai, J. P. Xie, Correlations between the fundamentals and applications of ultrasmall metal nanoclusters: Recent advances in catalysis and biomedical applications. *Nano Today* **36**, 101053 (2021).

15. M. F. Matus, H. Häkkinen, Understanding ligand-protected noble metal nanoclusters at work. *Nat. Rev. Mater.* **8**, 372–389 (2023).

16. K. Konishi, M. Iwasaki, M. Sugiuchi, Y. Shichibu, Ligand-based toolboxes for tuning of the optical properties of subnanometer gold clusters. *J. Phys. Chem. Lett.* **7**, 4267–4274 (2016).

17. K. L. D. M. Weerawardene, P. Pandeya, M. Zhou, Y. Chen, R. Jin, C. Aikens, Luminescence and electron dynamics in atomically precise nanoclusters with eight superatomic electrons. *J. Am. Chem. Soc.* **141**, 18715–18726 (2019).

18. N. A. Sakthivel, M. Shabaninezhad, L. Sementa, B. Yoon, M. Stener, R. L. Whetten, G. Ramakrishna, A. Fortunelli, U. Landman, A. Dass, The missing link: $Au_{191}(SPh\text{-}^{t}Bu)_{66}$ Janus nanoparticle with Molecular and bulk-metal-like properties. *J. Am. Chem. Soc.* **142**, 15799–15814 (2020).

19. M. McPartlin, R. Mason, L. Malatesta, Novel cluster complexes of gold(0)-gold(I). *J. Chem. Soc. D* **0** (1969), 334.

20. F. Fetzer, N. Pollard, N. C. Michenfelder, M. Strienz, A. N. Unterreiner, A. Z. Clayborne, A. Schnepf, $Au_{20}(^tBu_3P)_8$: A highly symmetric metalloid gold cluster in oxidation state 0. *Angew. Chem. Int. Ed.* **61**, e202206019 (2022).

21. Z. Lei, X. Wan, S.-F. Yuan, Z.-J. Guan, Q.-M. Wang, Alkynyl approach toward the protection of metal nanoclusters. *Acc. Chem. Res.* **51**, 2465–2474 (2018).

22. Y. Li, M. Zhou, Y. Song, T. Higaki, H. Wang, R. Jin, Double-helical assembly of heterodimeric nanoclusters into supercrystals. *Nature* **594**, 380–384.

23. N. Xia, J. Yuan, L. Liao, W. Zhang, J. Li, H. Deng, J. Yang, Z. Wu, Structural oscillation revealed in gold nanoparticles. *J. Am. Chem. Soc.* **142**, 12140–12145 (2020).

24. Zhang, C. Chen, W. Chuang, S. Chen, P. Size transformation of the $Au_{22}(SG)_{18}$ nanocluster and its surface-sensitive kinetics. *J. Am. Chem. Soc.* **142**, 11514–11520 (2020).

25. S.-F. Yuan, W.-D. Liu, C.-Y. Liu, Z.-J. Guan, Q.-M. Wang, Nitrogen donor protection for atomically precise metal nanoclusters. *Chem. Eur. J.* **28**, e202104445 (2022).

26. M. R. Narouz, K. M. Osten, P. J. Unsworth, R. W. Y. Man, K. Salorinne, S. Takano, R. Tomihara, S. Kaappa, S. Malola, C.-T. Dinh, J. D. Padmos, K. Ayoo, P. J. Garrett, M. Nambo, J. H. Horton, E. H. Sargent, H. Häkkinen, T. Tsukuda, C. M. Crudden, N-heterocyclic carbene-functionalized magic-number gold nanoclusters. *Nat. Chem.* **11**, 419–425 (2019).

27. M. R. Narouz, S. Takano, P. A. Lummis, T. I. Levchenko, A. Nazemi, S. Kaappa, S. Malola, G. Yousefalizadeh, L. A. Calhoun, K. G. Stamplecoskie, H. Häkkinen, T. Tsukuda, C. M. Crudden, Robust, highly luminescent Au_{13} superatoms protected by N-heterocyclic carbenes. *J. Am. Chem. Soc.* **141**, 14997–15002 (2019).

28. H. Shen, Z. Xu, M. S. A. Hazer, Q. Wu, J. Peng, R. Qin, S. Malola, B. K. Teo, H. Häkkinen, N. Zheng, Surface coordination of multiple ligands endows N-heterocyclic carbene-stabilized

gold nanoclusters with high robustness and surface reactivity. *Angew. Chem. Int. Ed.* **60**, 3752–3758 (2021).

29. R. W. Y. Man, H. Yi, S. Malola, S. Takano, T. Tsukuda, H. Hakkinen, M. Nambo, C. M. Crudden, Synthesis and characterization of enantiopure chiral bis NHC-stabilized edge-shared Au_{10} nanocluster with unique prolate shape. *J. Am. Chem. Soc.* **144**, 2056–2061 (2022).

30. V. K. Kulkarni, B. N. Khiarak, S. Takano, S. Malola, E. L. Albright, T. I. Levchenko, M. D. Aloisio, C.-T. Dinh, T. Tsukuda, H. Häkkinen, C. M. Crudden, N-heterocyclic carbene-stabilized hydrido Au_{24} nanoclusters: Synthesis, structure, and electrocatalytic reduction of CO_2. *J. Am. Chem. Soc.* **144**, 9000–9006 (2022).

31. J. M. Forward, J. P. Fackler, R. J. Staples, Synthesis and structural characterization of the luminescent gold(I) complex $[(MeTPA)_3AuI]I_3$. Use of $NaBPh_4$ as a phenyl-transfer reagent to form $[(MeTPA)AuPh](BPh_4)$ and (TPA)AuPh. *Organometallics* **14**, 4194–4198 (1995).

32. J. L. R. Williams, J. C. Doty, P. J. Grisdale, R. Searle, T. H. Regan, G. P. Happ, D. P. Maier, Boron photochemistry. I. Boron photochemistry. I. Irradiation of sodium tetraarylborates in aqueous solution. *J. Am. Chem. Soc.* **89**, 5153–5157 (1967).

33. X.-S. Han, X. Luan, H.-F. Su, J.-J. Li, S.-F. Yuan, Z. Lei, Y. Pei, Q.-M. Wang, Structure determination of alkynyl-protected gold nanocluster $Au_{22}(^tBuC{\equiv}C)_{18}$ and its thermochromic luminescence. *Angew. Chem. Int. Ed.* **59**, 2309–2312 (2020).

34. S. H. Strauss, The search for larger and more weakly coordinating anions. *Chem. Rev.* **93**, 927–942 (1993).

35. Y. Peng, G. Song, Combined microwave and ultrasound assisted Williamson ether synthesis in the absence of phase-transfer catalysts. *Green Chem.* **4**, 349–351 (2002).

36. M. Walter, J. Akola, O. Lopez-Acevedo, P. D. Jadzinsky, G. Calero, C. J. Ackerson, R. L. Whetten, H. Grönbeck, H. Häkkinen, A unified view of ligand-protected gold clusters as superatom complexes. *Proc. Natl. Acad. Sci. U.S.A.* **105**, 9157–9162 (2008).

37. D. M. P. Mingos, Structural and bonding patterns in gold clusters. *Dalton Trans.* **44**, 6680–6695 (2015).

38. H. Schmidbaur, A. Schier, Aurophilic interactions as a subject of current research: An up-date. *Chem. Soc. Rev.* **41**, 370–412 (2012).

39. X.-K. Wan, Z.-J. Guan, Q.-M. Wang, Homoleptic alkynyl-protected gold nanoclusters: $Au_{44}(PhC{\equiv}C)_{28}$ and $Au_{36}(PhC{\equiv}C)_{24}$. *Angew. Chem. Int. Ed.* **56**, 11494–11497 (2017).

40. F. Hu, Z.-J. Guan, G. Yang, J.-Q. Wang, J.-J. Li, S.-F. Yuan, G.-J. Liang, Q.-M. Wang, Molecular gold nanocluster Au_{156} showing metallic electron dynamics. *J. Am. Chem. Soc.* **143**, 17059–17067 (2021).

41. W.-D. Si, C. Zhang, M. Zhou, W.-D. Tian, Z. Wang, Q. S. Hu, K.-P. Song, L. Feng, X.-Q. Huang, Z.-Y. Gao, C.-H. Tung, D. Sun, Two triplet emitting states in one emitter: Near-infrared dual-phosphorescent Au_{20} nanocluster. *Sci. Adv.* **9**, eadg3587 (2023).

42. C. B. Aakeroy, D. L. Bryce, G. R. Desiraju, A. Frontera, A. C. Legon, F. Nicotra, K. Rissanen, S. Scheiner, G. Terraneo, P. Metrangolo, G. Resnati, Definition of the chalcogen bond (IUPAC Recommendations 2019). *Pure Appl. Chem.* **91**, 1889–1892 (2019).

43. H. Z. Yu, B. Rao, W. Jiang, S. Yang, M. Z. Zhu, The photoluminescent metal nanoclusters with atomic precision. *Coord. Chem. Rev.* **378**, 595–617 (2019).

44. C. Zhu, J. Xin, J. Li, H. Li, X. Kang, Y. Pei, M. Zhu, Fluorescence or phosphorescence? The metallic composition of the nanocluster kernel does matter. *Angew. Chem. Int. Ed.* **134**, e202205947 (2022).

45. M. Zhou, Z. Lei, Q. Guo, Q.-M. Wang, A. Xia, Solvent dependent excited state behaviors of luminescent gold(I)-silver(I) cluster with hypercoordinated carbon. *J. Phys. Chem. C* **119**, 14980–14988 (2015).

46. K. Pyo, V. D. Thanthirige, K. Kwak, P. Pandurangan, G. Ramakrishna, D. Lee, Ultrabright luminescence from gold nanoclusters: Rigidifying the Au(I)-thiolate shell. *J. Am. Chem. Soc.* **137**, 8244–8250 (2015).

47. P. J. Herbert, C. J. Ackerson, K. L. Knappenberger, Size-scalable near-infrared photoluminescence in gold monolayer protected clusters. *J. Phys. Chem. Lett.* **12**, 7531–7536 (2021).

48. Q. Li, M. Zhou, W. Y. So, J. Huang, M. Li, D. R. Kauffman, M. Cotlet, T. Higaki, L. A. Peteanu, Z. Shao, R. Jin, A monocuboctahedral series of gold nanoclusters: Photoluminescence origin, large enhancement, wide tunability, and structure−property correlation. *J. Am. Chem. Soc.* **141**, 5314–5325 (2019).

49. P. Luo, X. J. Zhai, S. Bai, Y. B. Si, X. Y. Dong, Y. F. Han, S. Q. Zang, Highly efficient circularly polarized luminescence from chiral Au_{13} clusters stabilized by enantiopure monodentate NHC ligands. *Angew. Chem. Int. Ed.* **62**, e202219017 (2023).

50. T. D. Green, C. Yi, C. Zeng, R. Jin, S. McGill, K. L. Knappenberger, Temperature-dependent photoluminescence of structurally-precise quantum-confined $Au_{25}(SC_8H_9)_{18}$ and $Au_{38}(SC_{12}H_{25})_{24}$ metal nanoparticles. *J. Phys. Chem. A* **118**, 10611–10621 (2014).

51. S. Takano, H. Hirai, T. Nakashima, T. Iwasa, T. Taketsugu, T. Tsukuda, Photoluminescence of doped superatoms $M@Au_{12}$ (M = Ru, Rh, Ir) homoleptically capped by $(Ph_2)PCH_2P(Ph_2)$: Efficient room-temperature phosphorescence from $Ru@Au_{12}$. *J. Am. Chem. Soc.* **143**, 10560–10564 (2021).

52. W. Ishii, Y. Okayasu, Y. Kobayashi, R. Tanaka, S. Katao, Y. Nishikawa, T. Kawai, T. Nakashima, Excited state engineering in Ag_{29} nanocluster through peripheral modification with silver(I) complexes for bright near-infrared photoluminescence. *J. Am. Chem. Soc.* **145**, 11236–11244 (2023).

53. R. Uson, A. Laguna, M. Laguna, D. A. Briggs, H. H. Murray, J. P. Fackler, (Tetrahydrothiophene)gold (I) or gold (III) complexes. *Inorg. Synth.* **26**, 85 (1989).

54. Rigaku Oxford Diffraction, CrysAlis^{Pro} Software system, version 1.171.40.25a, Rigaku Corporation, Oxford, UK (2018).

55. L. Palatinus, G. Chapuis, SUPERFLIP−A computer program for the solution of crystal structures by charge flipping in arbitrary dimensions. *J. Appl. Cryst.* **40**, 786–790 (2007).

56. G. M. Sheldrick, Crystal structure refinement with SHELXL. *Acta Crystallogr. C: Struct. Chem.* **71**, 3–8 (2015).

57. O. V. Dolomanov, L. J. Bourhis, R. J. Gildea, J. A. K. Howard, H. Puschmann, OLEX2: A complete structure solution, refinement and analysis program. *J. Appl. Cryst.* **42**, 339–341 (2009).

58. A. L. Spek, Structure validation in chemical crystallography. *Acta Crystallogr D Biol Crystallogr.* **65**, 148–155 (2009).

59. A. L. Spek, PLATONSQUEEZE: A tool for the calculation of the disordered solvent contribution to the calculated structure factors. *Acta Crystallogr. C Struct. Chem.* **71**, 9–18 (2015).

60. M. J. Frisch, G. W. Trucks, H. B. Schlegel, G. E. Scuseria, M. A. Robb, J. R. Cheeseman, G. Scalmani, V. Barone, G. A. Petersson, H. Nakatsuji, X. Li, M. Caricato, A. V. Marenich, J. Bloino, B. G. Janesko, R. Gomperts, B. Mennucci, H. P. Hratchian, J. V. Ortiz, A. F. Izmaylov, A. F. Sonnenberg, J. L. Williams-Young, D. Ding, F. Lipparini, F. Egidi, F. Goings, J. Peng, B. Petrone, A. Henderson, T. Ranasinghe, D. Zakrzewski, V. G. Gao, J. Rega, N. Zheng, G. Liang, W. Hada, M. Ehara, M. Toyota, K. Fukuda, R. Hasegawa, J. Ishida, M. Nakajima, T. Honda, Y. Kitao, O. Nakai, H. Vreven, T. Throssell, K. Montgomery Jr., J. A. Peralta, J. E. Ogliaro, F. Bearpark, M. J. Heyd, J. J. Brothers, E. N. Kudin, K. N. Staroverov, V. N. Keith, T. A. Kobayashi, R. Normand, J. Raghavachari, K. Rendell, A. P. Burant, J. C. Iyengar, S. S. Tomasi, J. Cossi, M. Millam, J. M. Klene, M. Adamo, C. Cammi, R. Ochterski, J. W. Martin, R. L. Morokuma, K. Farkas, O. Foresman, J. B. Fox, D. J. Gaussian, Gaussian 16, Rev. B.01, Gaussian, Inc., Wallingford, CT (2016).

61. M. J. Frisch, J. A. Pople, J. S. Binkley, Self-consistent molecular orbital methods 25. Supplementary functions for Gaussian basis sets. *J. Chem. Phys.* **80**, 3265–3269 (1984).

62. Becke, A. D. Density-functional exchange-energy approximation with correct asymptotic behavior. *Phys. Rev. A* **38**, 3098–3100 (1988).

63. P. J. Hay, W. R. Wadt, Ab initioeffective core potentials for molecular calculations. Potentials for K to Au including the outermost core orbitals. *J. Chem. Phys.* **82**, 299–310 (1985).

64. T. Lu, F. Chen, Multiwfn: A multifunctional wavefunction analyzer. *J. Comput. Chem.* **33**, 580–592 (2012).
